# Neuromodulation and Copper Chelation Reverse Sleep Fragmentation-Aggravated Myocardial Ischemia–Reperfusion Injury by Targeting NET-Induced Endothelial Cuproptosis

**DOI:** 10.34133/research.1266

**Published:** 2026-05-08

**Authors:** Pilong Shi, Yuetong Sha, Xue Guan, Jiawei Wu, Jing Yang, Dongyu Min, Cong Wang, Yonggang Cao, Xinran Wang, Yuandong Qiao, Hongli Sun

**Affiliations:** ^1^ Department of Pharmacology, Harbin Medical University, Heilongjiang 163319, China.; ^2^ Experimental Center of Traditional Chinese Medicine, the Affiliated Hospital of Liaoning University of Traditional Chinese Medicine, Liaoning 110000, China.; ^3^ Key Laboratory of Basic Research and Health Management on Chronic Diseases in Heilongjiang Province, Harbin Medical University, Heilongjiang 163319, China.; ^4^ Department of Medical Morphology, Harbin Medical University, Heilongjiang 163319, China.; ^5^ Key Laboratory of Frigid Zone Exercise Health Research and Translation in Heilongjiang Province, Harbin Medical University, Heilongjiang 163319, China.; ^6^ Department of Cardiology, Daqing People’s Hospital, Heilongjiang 163319, China.; ^7^ Key Laboratory of Preservation of Human Genetic Resources and Disease Control in China, Harbin Medical University, Heilongjiang 150081, China.; ^8^ Department of Medical Genetics, Harbin Medical University, Heilongjiang 150081, China.

## Abstract

This study sought to investigate the link between sleep disorders and cardiac microvascular injury in myocardial ischemia–reperfusion injury (MI/RI) mice. Mice were subjected to a sleep deprivation protocol within a designated chamber. During the light phase (ZT0 to ZT12), a sweep bar moved across the cage floor at 2-min intervals, whereas it remained static throughout the dark phase (ZT12 to ZT24), with this routine maintained for 16 weeks. Subsequently, an MI/RI model was established to assess the extent of cardiac microvascular injury, and the underlying mechanisms were explored via proteomic analyses. It was demonstrated that 16 weeks of sleep fragmentation (SF) intensified cardiac microvascular damage in MI/RI mice. From a mechanistic perspective, SF was found to induce sympathetic hyperactivity, elevate plasma epinephrine levels, and consequently facilitate neutrophil chemotaxis and the generation of neutrophil extracellular traps (NETs). Moreover, the findings revealed that NETs suppressed Atox1 expression, impaired ATP7A-mediated copper transport, and contributed to copper accumulation within cardiac microvascular endothelial cells (CMECs) and oxidative stress. This copper overload further augmented cuproptosis, while these pathological alterations were shown to be reversible through sympathetic denervation, vagal electrical stimulation (ES), targeted delivery of copper chelators, or the inhibition of NETs. Overall, our data established that SF exacerbated MI/RI by promoting copper overload in CMECs. This study elucidated a molecular pathway through which sleep disturbances aggravated cardiac microvascular damage and suggested prospective targets for treatment strategies.

## Introduction

Sleep has been recognized as an essential component in maintaining cardiovascular integrity [1]. A significant body of research indicates that sleep disruptions are associated with higher rates of illness and death due to coronary heart disease [2]. Experimental research utilizing animal models has substantiated the detrimental impact of sleep disorders on myocardial injury [3,4]. A rigorous and comprehensive systematic review revealed that individuals with curtailed sleep durations face a statistically significant increase in the likelihood of developing coronary heart disease [5]. Nevertheless, the molecular mechanisms underpinning these associations remain incompletely characterized and demand further in-depth exploration.

Communication between the brain and heart has been recognized as essential for maintaining physiological homeostasis. The functionality of the brain–heart axis is modulated by a range of factors, among which sleep represents a pivotal component. Adequate sleep is indispensable for sustaining well-being [6], whereas insufficient or disrupted sleep has been linked to elevated risk of myocardial infarction, independent of genetic predisposition and other conventional risk determinants [7]. Recent investigations have highlighted intricate neural and immune-related pathways through which the sleeping brain governs biological mechanisms implicated in cardiovascular pathology. For instance, sleep-modulating signals originating from the hypothalamus influence immune cell generation, thereby impacting atherosclerotic progression [8]. Nonetheless, it has yet to be fully clarified whether sleep alters cardiovascular injury or disease states via interoceptive heart-to-brain communication and whether such sleep-dependent modifications affect brain-driven regulation of inflammatory responses to cardiac insult.

Acute myocardial infarction and coronary artery lesion [9–12] continues to represent a contributor to global mortality and morbidity. Although early reperfusion strategies, such as percutaneous coronary intervention, are capable of rescuing ischemic myocardium and enhancing survival outcomes, the abrupt re-establishment of blood flow may precipitate myocardial ischemia–reperfusion injury (MI/RI) [13]. The biological process driving MI/RI involves diverse pathways and incorporates fundamental molecular alterations, including mitochondrial dysfunction, oxidative stress, intracellular Ca^2+^ accumulation, and inflammatory activation [14–16]. Ischemia-induced deprivation of oxygen and nutrients results in bioenergetic exhaustion, buildup of metabolic waste, and deterioration of cellular function. Upon reperfusion, tissue damage is intensified through a series of harmful processes: The excessive generation of reactive oxygen species (ROS) compromises endothelial integrity, elevates oxidative stress, impairs vascular homeostasis, and facilitates inflammation, apoptotic signaling, and increased vascular permeability. Furthermore, reperfusion initiates a robust inflammatory cascade marked by immune cell recruitment, pro-inflammatory cytokine release, and neutrophil (NE) accumulation, all of which collectively exacerbate myocardial injury. Among the various cellular participants in MI/RI, NEs have been identified as key contributors to the progression of damage [17].

Neutrophil extracellular traps (NETs) represent extracellular lattice-like structures that are discharged during NETosis, a self-destructive inflammatory mechanism of NE demise [18]. NETs are composed of a DNA framework intertwined with various proteins that exhibit bactericidal activity and enhance membrane permeability, such as citrullinated histone 3 (citH3), myeloperoxidase (MPO), and NE elastase [19,20]. MI/RI is characterized as a multifactorial process in which NEs facilitate the recruitment of additional inflammatory cells and secrete pathogenic enzymes, such as matrix metalloproteinases, chemotactic mediators, and MPO, that further intensify myocardial damage within infarcted tissue [21,22]. Additionally, studies have demonstrated that NET formation exacerbates enhanced inflammation-related damage following a myocardial infarction [23]. Evidence suggests that NETs promote platelet adhesion and aggregation, thereby forming a structural scaffold that supports thrombus development [24]. They have also been implicated in the pathogenesis of acute myocardial infarction through the enhancement of fibrin accumulation and facilitation of red blood cell agglutination, ultimately contributing to thrombus progression [25]. These findings suggest that NETs are integral to the pathophysiological landscape of MI/RI and that therapeutic approaches targeting NETs may offer clinical benefit.

Myocardial injury has been associated with disturbances in ion homeostasis. Copper equilibrium serves a fundamental function in sustaining numerous physiological processes in organisms [26], including mitochondrial energy metabolism [27], redox regulation [28], tyrosine and neurotransmitter biosynthesis [29], and extracellular matrix remodeling [30]. A substantial body of evidence has indicated that copper deficiency may result in enhanced collagen accumulation and myocardial fibrosis [31], compromised antioxidant defense mechanisms accompanied by heightened vulnerability to oxidative stress [32], and altered angiogenic activity [33]. Additionally, the harmful consequences of copper overload have also been well-documented. Intracellular copper excess has been shown to initiate apoptotic signaling through both intrinsic and extrinsic pathways [34]. Moreover, excessive copper levels trigger cuproptosis, a newly discovered form of cellular death that depends on copper presence, characterized by iron–sulfur cluster protein reduction, decreased lipoylated protein quantities, and enhanced DLAT oligomer formation [35]. Nevertheless, the specific contribution of elevated copper levels or cuproptosis pathways to cardiovascular disease progression remains largely unexplored.

Sympathetic hyperactivity has been recognized as a prominent pathological hallmark in sleep disorders and heart disease [36–40]. Nonetheless, the association between sympathetic signaling and the maintenance of endothelial copper homeostasis has yet to be fully established. To explore how sleep fragmentation (SF) influences endothelial copper metabolism and its underlying molecular mechanisms, a series of murine models were employed: (a) an MI/RI model exacerbated by chronic sleep fragmentation (SF + MI/RI)‌ to replicate SF-aggravated cardiac microvascular injury, ‌(b) an in vitro NE-based model exposed to epinephrine (EPI)‌ to dissect sympathetic-induced NE chemotaxis and NET formation, and ‌(c) a copper overload model in cardiac microvascular endothelial cells (CMECs) triggered by NETs‌ to directly link NETosis to intracellular copper dysregulation and cuproptosis. These models were also utilized to evaluate the therapeutic effectiveness of interventions targeting endothelial copper accumulation under ischemia–reperfusion (I/R) conditions complicated by SF. The findings demonstrated that modulation of endothelial copper balance by sympathetic neural activity constitutes a mechanistic link in brain–heart communication.

## Results

### Chronic SF exacerbated the injury of cardiac microvascular endothelium in the MI/RI model

**Fig. 1.**
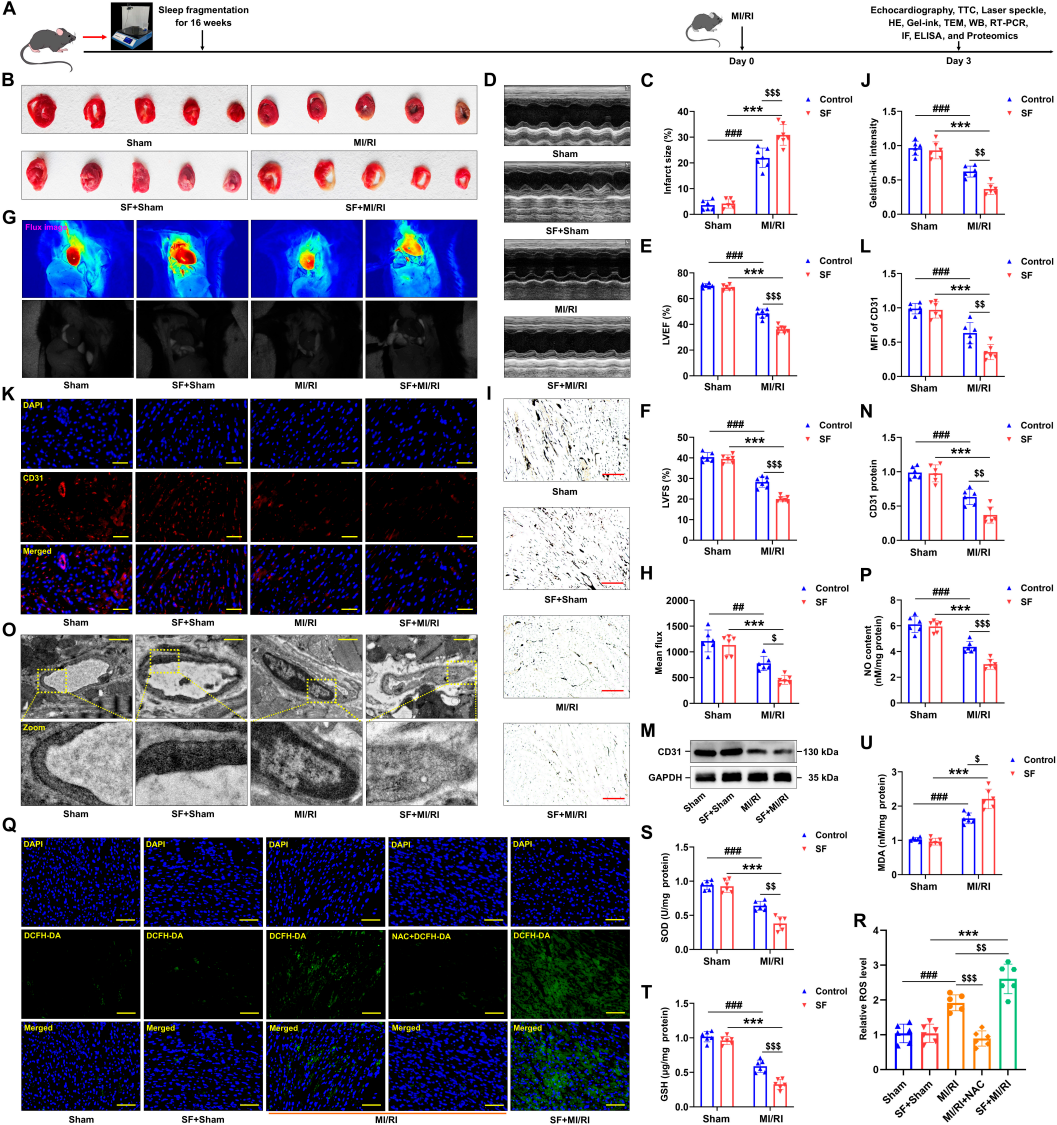
Chronic SF aggravated the cardiac microvascular injury in the MI/RI model. (A) Scheme of the time axis representing the design of the animal study. (B and C) Detection of infarct areas in cardiac tissue sections with TTC staining (*n* = 6 mice for Sham and SF + Sham group, *n* = 7 mice for MI/RI and SF + MI/RI group). (D) Representative M-mode echocardiographic changes. (E and F) Left ventricular ejection fraction (LVEF) and left ventricular fractional shortening (LVFS) were assessed by echocardiography in mice with SF and control mice after MI/RI (*n* = 6 mice for Sham and SF + Sham group, *n* = 7 mice for MI/RI and SF + MI/RI group). (G) The LASCA technique was used to assess the flow velocity in the infarct area of various groups. (H) The mean flux was qualified (*n* = 6 mice per group). (I and J) After MI/RI, hearts were injected with gelatin–ink, and samples were observed via microscope (*n* = 6 mice per group). (K and L) CD31 was quantified by calculating the mean fluorescence intensity. MFI, mean fluorescence intensity (*n* = 6 per group). (M and N) Validation of the levels of CD31 by Western blotting (*n* = 6 per group). (O) After MI/RI, the ultrastructure of microvessels was observed via the electron microscope (*n* = 6 per group). (P) Quantification of NO (*n* = 6 per group). (Q and R) Quantitative analysis of ROS (labeled by DCFH-DA) (*n* = 6 per group). NAC, N-acetylcysteine. (S to U) Levels of superoxide dismutase (SOD), glutathione (GSH), and malondialdehyde (MDA) in different group (*n* = 6 per group). Data are presented as mean ± SD. ^##^*P* < 0.01 versus Sham group; ^###^*P* < 0.001 versus Sham group; ^***^P < 0.001 versus SF + Sham group; ^$^*P* < 0.05 versus MI/RI group; ^$$^*P* < 0.01 versus MI/RI group; ^$$$^*P* < 0.001 versus MI/RI group. Scale bars, 50 μm (I and Q), 25 μm (K), and 1 μm (O).

To explore the impact of sleep disturbances on cardiac microvascular injury in MI/RI model, C57BL/6 mice were subjected to chronic SF for 16 weeks prior to the induction of the MI/RI model (Fig. 1A). Mice exposed to SF showed significantly larger infarct sizes post-MI/RI (Fig. 1B and C) and markedly worse cardiac function than controls (Fig. 1D to F), consistent with prior findings [41]. Moving forward, we will conduct a more in-depth investigation into the specific effects of SF on cardiac microvascular function within the MI/RI experimental model. Blood flow serves as a pivotal indicator of infarcted myocardial regions. Therefore, laser speckle contrast analysis (LASCA) was employed to analyze flow velocity in affected areas, thereby offering insight into circulatory alterations. A pronounced obstruction of blood flow was identified in SF-treated mice, as demonstrated in Fig. 1G and H. Additionally, as presented in Fig. 1I and J, I/R-injured hearts displayed linear and irregular perfusion defects within the microvasculature, which were further exacerbated by SF exposure. Subsequent assessments revealed a substantial reduction in both blood flow and microvessel density on days 3 and 7 post-I/R in the SF group relative to controls (Fig. S1A to D). Disruption of integrity and barrier function of microvasculature are regarded as a fundamental factor in the onset of microcirculatory impairment. Notably, immunofluorescence analysis showed that CD31 signal intensity decreased to approximately 70% of baseline following I/R injury, and was further diminished to roughly 40% with SF treatment (Fig. 1K and L). This trend was consistent with Western blot results, which demonstrated that CD31 protein expression was substantially suppressed by MI/RI and further reduced in SF-exposed mice (Fig. 1M and N). In line with these findings, ultrastructural changes in microvasculature were examined by electron microscopy (EM). As illustrated in Fig. 1O, hearts subjected to I/R displayed swollen endothelial cells and irregular vessel walls compared to the sham group. Structural impairment of microvessels was further intensified under SF conditions. Research indicated that enhanced nitric oxide (NO) release prior to ischemia offers protection against MI/RI [42]. Our ‌experimental findings revealed that SF caused a significant reduction in NO production (Fig. 1P and Fig. S1E). Moreover, SF intensified the I/R-induced dramatic rise in ROS, and malondialdehyde (MDA), and the SF group exhibited lower glutathione (GSH) levels and superoxide dismutase (SOD) activity (Fig. 1Q to U). Collectively, these data confirm the occurrence of cardiac microvascular injury in SF-treated myocardium, although the underlying molecular mechanisms remain to be clarified.

### Chronic SF promoted NET formation of MI/RI model mice

**Fig. 2.**
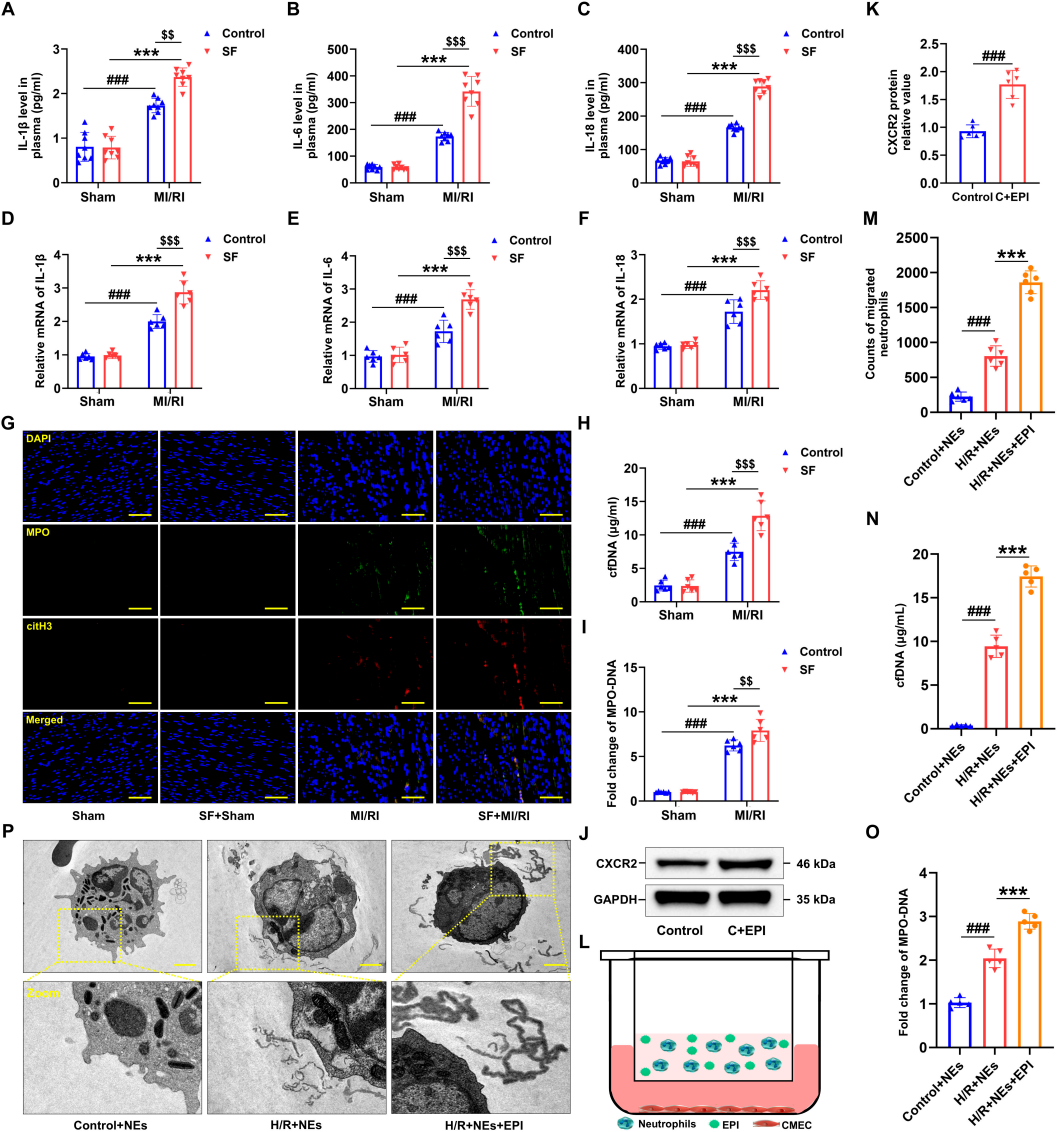
Chronic SF exacerbated the inflammatory response and promoted the formation of NETs in MI/RI mice. (A to C) Plasma concentrations of IL-1β, IL-6, and IL-18 in mice were measured by ELISA (*n* = 8 per group). (D to F) qRT-PCR assays were performed to determine mRNA levels of IL-1β, IL-6, and IL-18 in the hearts of MI/RI mice with SF (*n* = 6 per group). (G) Representative images of immunofluorescence staining of NETs in myocardial tissues from Sham, SF + Sham, MI/RI, and SF + MI/RI groups (green: MPO, red: citH3, blue: DAPI) (*n* = 6 per group). (H) Cell-free DNA (cfDNA) of each group (*n* = 6 per group). (I) The MPO–DNA complex relative levels were assessed in mice (*n* = 6 per group). Data are presented as mean ± SD. ^###^*P* < 0.001 versus Sham group; ^***^P < 0.001 versus SF + Sham group; ^$$^*P* < 0.01 versus MI/RI group; ^$$$^*P* < 0.001 versus MI/RI group. (J and K) Western blotting was used to analyze the expression of CXCR2 in NEs after treatment with EPI for 6 h (*n* = 6 per group). Data are presented as mean ± SD. ^###^*P* < 0.001 versus Control group. C + EPI, Control + EPI. (L) Schematic diagram of coculture of EPI-induced NEs and CMECs exposed to H/R. CMECs were treated with hypoxia for 12 h and reoxygenation for 24 h and then cocultured with NEs for 6 h. (M) Statistical analysis of the number of migrated NEs after coculture with H/R-induced CMECs (*n* = 6 independent experiments). (N and O) The cfDNA and MPO–DNA complex levels were assessed (*n* = 5 independent experiments). (P) After coculture, the ultrastructure of NEs was observed via the electron microscope. Data are presented as mean ± SD. ^###^*P* < 0.001 versus Control + NEs group; ^***^P < 0.001 versus H/R + NEs group. Scale bars, 50 μm (G) and 2.5 μm (P). NEs, neutrophils; EPI, epinephrine.

MI/RI is marked by a variety of pathological processes, with the inflammatory response standing out as a key feature [43]. Both inflammation and the infiltration of inflammatory cells serve as recognized hallmarks of MI/RI pathology [44,45]. Nevertheless, the mechanistic association between SF and inflammation remains insufficiently elucidated. As anticipated, elevated circulating concentrations of interleukin-1β (IL-1β), IL-6, and IL-18 were detected in MI/RI mice, with these levels being further enhanced by SF exposure (Fig. 2A to C). Additionally, mRNA expression of IL-1β, IL-6, and IL-18 in cardiac tissue revealed that SF exacerbated the inflammatory profile within the myocardium following MI/RI (Fig. 2D to F). NEs, when activated, can undergo NETosis, a process involving the release of NETs [46]. NETs are known to connect inflammation and thrombosis and have been implicated in worsening MI/RI outcomes [47]. To determine whether NETs participate in SF-related myocardial injury, the extent of NET formation in cardiac tissue was examined in SF-exposed mice after MI/RI. Compared to MI/RI mice, SF pretreatment increased MPO and citH3 immunofluorescence colocalization signal (Fig. 2G), indicative of NET accumulation, as demonstrated by confocal microscopy. Furthermore, quantification of cell-free DNA (cfDNA) and MPO-DNA levels showed that SF-treated MI/RI mice exhibited markedly elevated concentrations of both markers relative to MI/RI alone (Fig. 2H and I), supporting a link between NETosis and SF. Collectively, these results indicate that NET accumulation induced by SF substantially contributes to the advancement of MI/RI and is linked to increased disease severity.

Sympathetic nervous system overactivation is widely acknowledged as a frequent pathophysiological trait of sleep disturbances. The current data demonstrated that relative to control mice, animals exposed to SF exhibited markedly elevated plasma EPI levels, the main neurotransmitter released at sympathetic nerve endings, while myocardial EPI concentrations remained unchanged (Fig. S2A and B). Consequently, excessive sympathetic activity may facilitate the recruitment of NEs to injured myocardial tissue via chemotaxis. This hypothesis was supported by increased CXCR2 protein expression in NEs following EPI exposure (Fig. 2J and K), suggesting that intensified inflammatory responses are likely to occur in the myocardium of mice subjected to combined SF and I/R insults. To further investigate the role of EPI in NE chemotaxis and NET generation, EPI-stimulated NEs were cocultured with hypoxia–reoxygenation (H/R)-treated CMECs (Fig. 2L). The results indicated a significant enhancement of NE chemotactic activity following EPI treatment (Fig. 2M). Moreover, elevated levels of cfDNA, MPO–DNA, and ultrastructural changes identified by EM collectively confirmed the EPI-mediated promotion of NET formation (Fig. 2N to P). Collectively, these findings indicate that EPI robustly activates NE chemotaxis and stimulates NET generation, highlighting a contributory role of sympathetic signaling in the exacerbation of MI/RI.

### Atox1 was a pivotal regulator of microvascular dysfunction in MI/RI mice

**Fig. 3.**
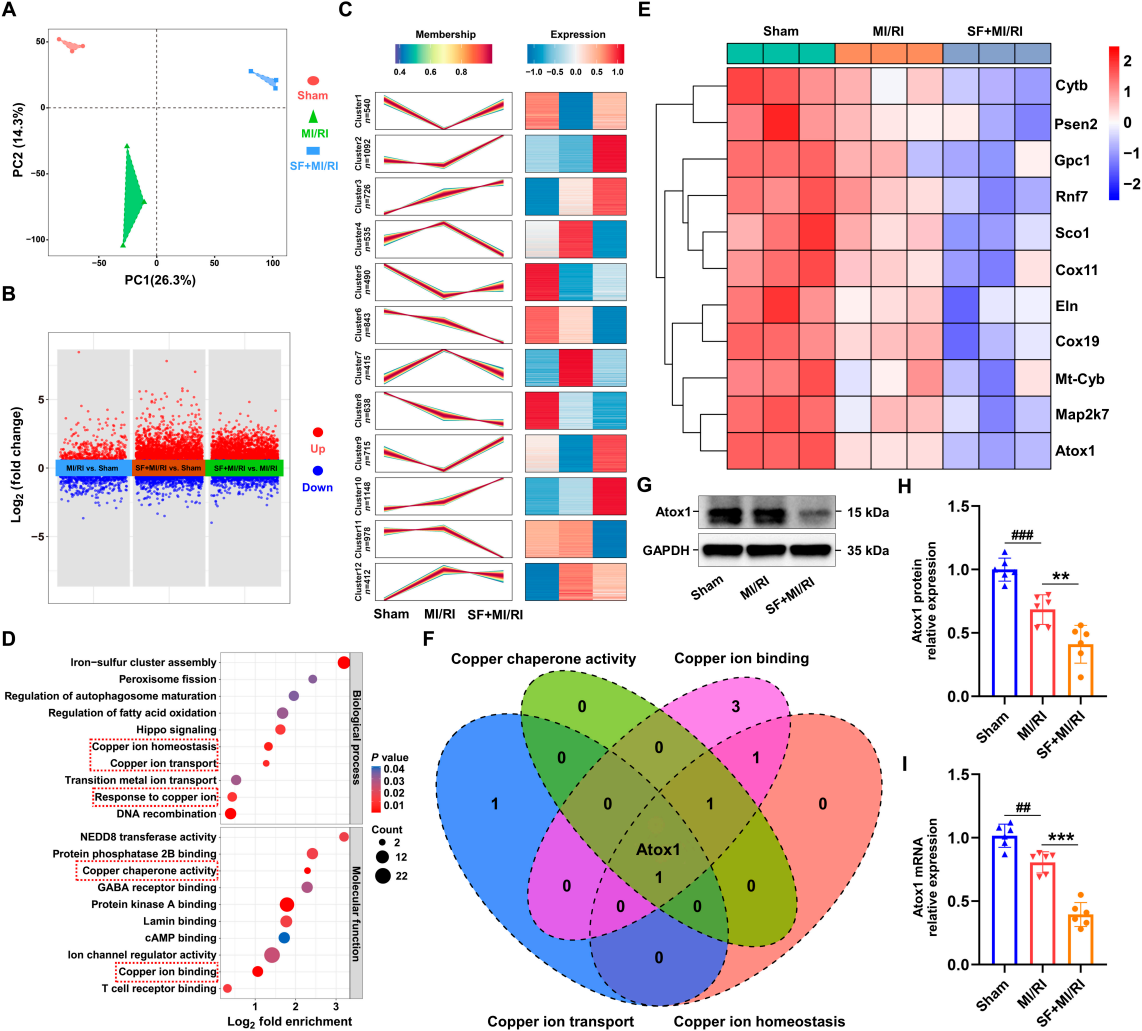
Chronic SF exacerbated the down-regulation of Atox1 induced by MI/RI. (A) Principal components analysis of proteomics. (B) Difference scatterplot of proteomics. (C) Expression pattern cluster analysis diagram. (D) Analysis of GO function. (E) Heatmap of proteomics. (F) Venn diagram illustrating the overlap of key protein (Atox1) related to copper overload. (G and H) Western blotting was used to observe the expression of Atox1 in Sham, MI/RI, and SF + MI/RI groups (*n* = 6 per group). (I) The mRNA levels of Atox1 in each group were measured by qRT-PCR (*n* = 6 per group). β-Actin was used as an internal control. Data are presented as mean ± SD. ^##^*P* < 0.01 versus Sham group; ^###^*P* < 0.001 versus Sham group; ^**^P < 0.01 versus MI/RI group; ^***^P < 0.001 versus MI/RI group.

To elucidate the underlying mechanisms through which chronic SF exacerbates cardiac microvascular injury in MI/RI, a comprehensive quantitative proteomic analysis was performed. Myocardial tissue samples were specifically harvested for this analysis. CMECs, which are the primary site of injury in our model, are integral components of the myocardial tissue. Analyzing the whole myocardial proteome provides a holistic view of the cellular and molecular landscape, capturing changes not only within CMECs but also in their interactions with surrounding cardiomyocytes and the interstitial environment. As illustrated in Fig. 3A and B, principal component analysis alongside scatterplots depicted the variations in protein expression. Through quantitative proteomic profiling, a total of 9,305 proteins were detected. To pinpoint proteins with substantial alterations, log_2_ transformation was applied to the relative expression levels. In total, 8,532 proteins underwent clustering analysis to identify expression patterns, as demonstrated in Fig. 3C. Building upon the findings derived from Figs. 1 and 2, and taking into account the differential expression patterns of proteins across clusters, we have prioritized clusters 6 and 8—characterized by their consistent and robust expression same trends—for aggravated MI/RI. Gene Ontology (GO) enrichment analysis was then conducted for clusters 6 and 8 to characterize the biological process involved, revealing pronounced changes in pathways such as copper ion homeostasis, copper ion transport, response to copper ion, copper chaperone activity, and copper ion binding, with results shown in Fig. 3D. Notably, quantification of myocardial copper levels indicated a marked elevation in mice exposed to SF following MI/RI (Fig. S3A). Additionally, the initiation of cuproptosis was supported by the observed up-regulation of DLAT oligomers in the SF + MI/RI group (Fig. S3B and C). Moreover, reduced expression level of FDX1 was detected (Fig. S3D and E), implying the occurrence of severe copper overload in the myocardium under SF + MI/RI conditions. A heat map displaying proteins associated with these biological functions is provided in Fig. 3E. Integrated analysis of datasets relating to copper ion identified Atox1, highlighting its pivotal role in the SF-induced modulation of MI/RI (Fig. 3F). Based on the cumulative evidence, it was proposed that SF-induced down-regulation of Atox1 might be a key driver of cuproptosis during MI/RI. Corroborating the proteomic findings, both protein and mRNA levels of Atox1 were substantially diminished following MI/RI, with further suppression observed upon SF intervention (Fig. 3G to I). These observations suggest that cuproptosis may occur in the hearts of SF + MI/RI mice, thereby intensifying the damage to CMECs.

### NETs contributed to cuproptosis in CMECs

**Fig. 4.**
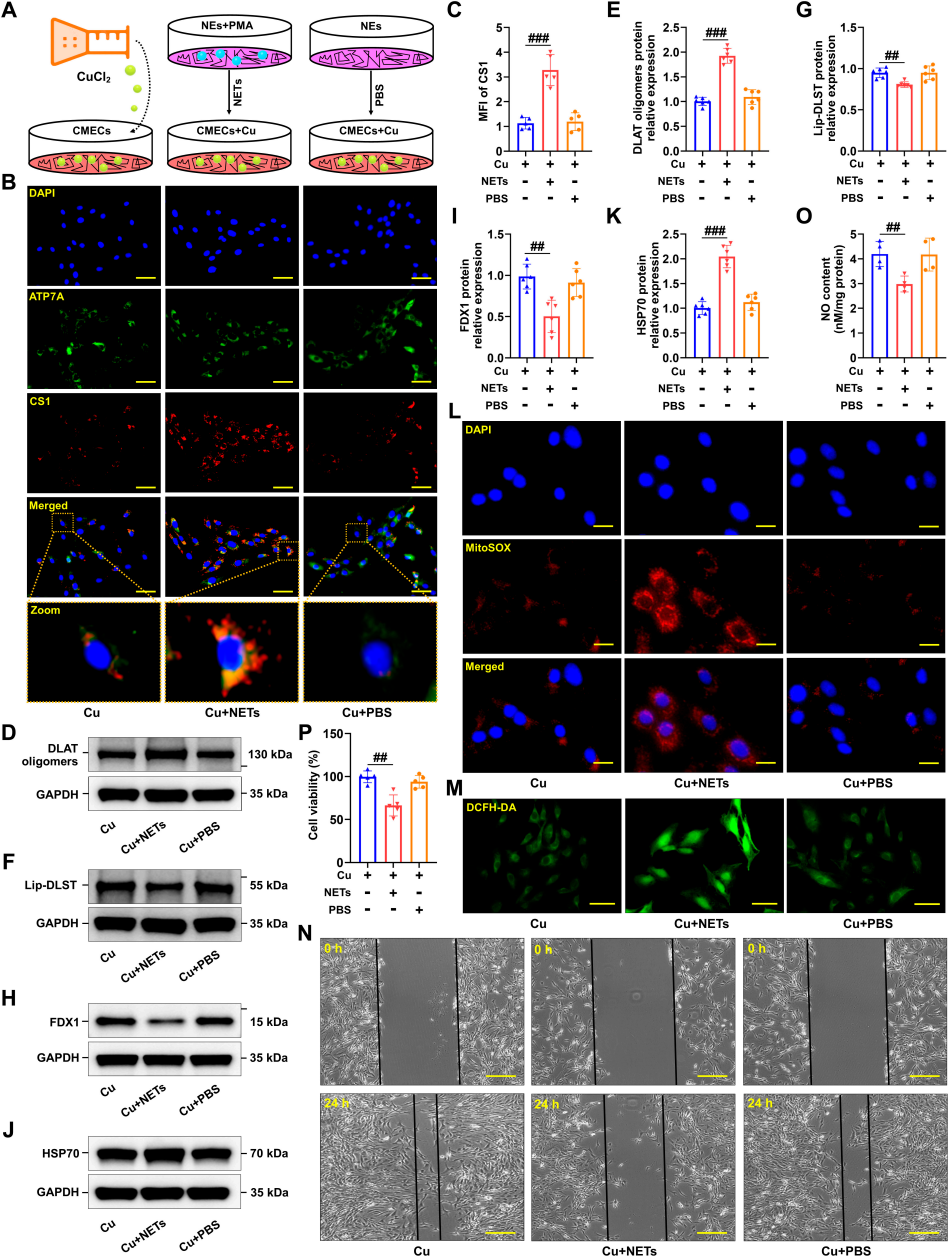
NETs induced cuproptosis in CMECs. (A) Schematic diagram of the experimental groups in the cell model. CMECs were treated with 10 μM CuCl_2_, NETs, or PBS for 24 h. NEs were stimulated by 50 nM PMA for 4 h to induce NETs. (B) Representative images of copper overload and ATP7A localization (red: CS1, green: ATP7A, blue: DAPI). CS1, Coppersensor-1, indicated intracellular copper ions. (C) CS1 staining was quantified by calculating the mean fluorescence intensity (*n* = 5 independent experiments). (D to K) Validation of the levels of DLAT oligomers, lipoylated proteins, iron–sulfur cluster proteins, and HSP70 by Western blotting after treating CMECs with 10 μM CuCl_2_ in the presence of NETs or PBS for 24 h (*n* = 6 per group). (L and M) Representative fluorescence images of mitochondrial and intracellular ROS production in CMECs induced by NETs (*n* = 6 per group). (N) The scratch test was used to detect the migration ability of CMECs (*n* = 6 per group). (O) Statistical analysis of NO content (*n* = 4 per group). (P) Cell viability was evaluated using the CCK-8 assay (*n* = 5 per group). Data are presented as mean ± SD. ^##^*P* < 0.01 versus CMECs + Cu group; ^###^*P* < 0.001 versus CMECs + Cu group. Scale bars, 25 μm (B and M), 10 μm (L), and 50 μm (N).

Firstly, to ascertain whether EPI directly modulates Atox1 expression and cuproptosis in CMECs, CMECs were incubated with EPI in conjunction with CuCl_2_ for 24 h. The findings revealed that EPI exerted no discernible impact on the protein levels of Atox1, DLAT oligomers, Lip-DLST, and FDX1 (Fig. S4A to H). Subsequently, the involvement of NETs in cuproptosis and MI/RI was explored. To address this hypothesis, an in vitro model was established wherein CMECs were treated with CuCl_2_ to elucidate whether and in what manner NETs influence copper transport (Fig. 4A). Coppersensor-1 (CS1), a selective and inducible fluorescent probe, was employed to visualize intracellular copper dynamics. Immunofluorescence analysis demonstrated that CuCl_2_ exposure initiated intracellular copper transport and triggered ATP7A mobilization for vesicular copper trafficking. Moreover, NET stimulation was observed to markedly elevate intracellular copper levels and obstruct copper transport, as evidenced by enhanced colocalization between ATP7A and CS1 fluorescence (Fig. 4B and C), along with significant activation of cuproptosis (Fig. 4D to K). Subsequently, NETs were observed to significantly enhance ROS generation in CMECs exposed to copper (Fig. 4L and M). Additionally, wound healing assays revealed a substantial reduction in migration distance following NET exposure (Fig. 4N), while NO levels were also diminished (Fig. 4O). Cell Counting Kit-8 (CCK-8) assay results further corroborated that NET treatment led to a decline in CMEC viability (Fig. 4P). Collectively, these observations indicate that NETs markedly promote intracellular copper overload and the activation of cuproptosis, suggesting a pivotal role for NETs in mediating endothelial copper dysregulation.

### NETs promoted CMEC cuproptosis by inhibiting Atox1 expression

**Fig. 5.**
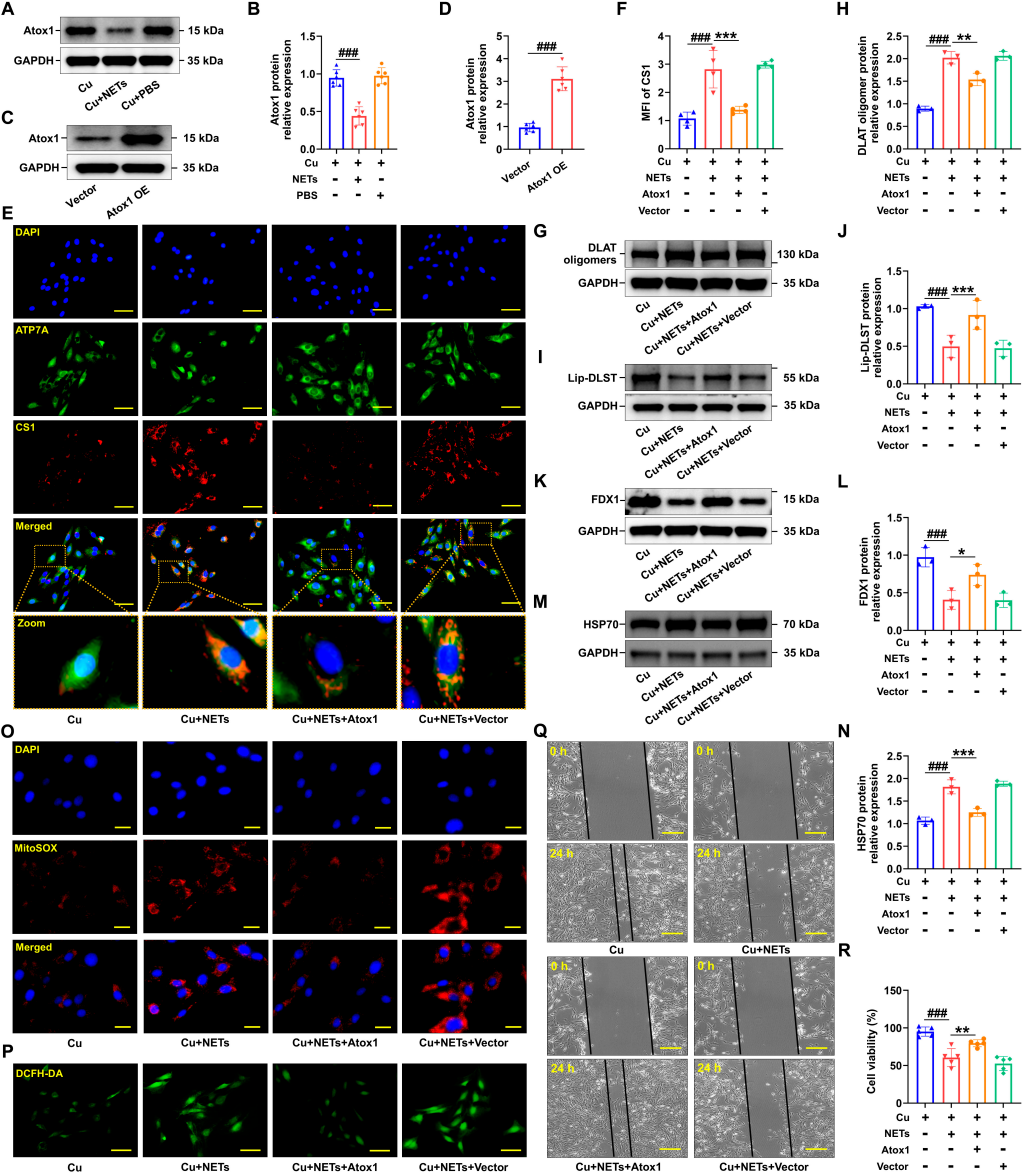
Overexpression of Atox1 attenuated cuproptosis induced by NETs in CMECs. (A and B) Western blotting and quantification of Atox1 in CMECs in Cu, Cu + NETs, or Cu + PBS group for 24 h (*n* = 6 independent experiments). (C) Validation of the overexpression (OE) of Atox1 in CMECs. (D) Statistical analysis of Atox1 overexpression (*n* = 6 independent experiments). Data are presented as mean ± SD. ^###^*P* < 0.001 versus Vector group. (E) Representative images of copper overload and ATP7A localization. (F) CS1 staining was quantified by calculating the mean fluorescence intensity (*n* = 4 per group). (G to N) Validation of the levels of DLAT oligomers, lipoylated proteins, iron–sulfur cluster proteins, and HSP70 by Western blotting after Vector- and Atox1-overexpressing CMECs with 10 μM CuCl_2_ in the presence of NETs for 24 h (*n* = 3 per group). (O and P) Representative fluorescence images of mitochondrial and intracellular ROS production in NET-induced CMECs after treatment with Atox1 overexpression (*n* = 6 per group). (Q) The scratch test was used to detect the migration ability of CMECs (*n* = 6 per group). (R) Cell viability was evaluated using the CCK-8 assay (*n* = 5 per group). Data are presented as mean ± SD. ^###^*P* < 0.001 versus CMECs + Cu group; ^*^P < 0.05 versus CMECs + Cu + NETs group; ^**^P < 0.01 versus CMECs + Cu + NETs group; ^***^P < 0.001 versus CMECs + Cu + NETs group. Scale bars, 25 μm (E and P), 10 μm (O), and 50 μm (Q).

Atox1 was previously identified as a component of the intracellular copper ion transport machinery, and its expression was observed to be suppressed in CMECs subjected to NET treatment (Fig. 5A and B). It was postulated that the down-regulation of Atox1 constituted a principal factor contributing to the NET-induced impairment of intracellular copper trafficking. Therefore, CMECs engineered to overexpress Atox1 (Fig. 5C and D) were utilized, revealing that Atox1 overexpression markedly enhanced intracellular copper transport and mitigated copper accumulation following NET exposure (Fig. 5E and F). Moreover, Atox1 overexpression markedly diminished DLST oligomer formation (Fig. 5G and H), restored lipoylated protein levels (Fig. 5I and J) and iron–sulfur cluster protein content (Fig. 5K and L), and reduced heat shock protein 70 (HSP70) expression (Fig. 5M and N), thereby indicating a reversal of cuproptosis and oxidative stress (Fig. 5O and P and Fig. S5A to C), improvement in CMEC functionality (Fig. 5Q), and an elevation in cellular viability (Fig. 5R). Moreover, knockdown of Atox1 exacerbated cuproptosis and dysfunction in CMECs exposed to NETs (Fig. S6A to H). These findings suggest that NETs generated by sympathetic hyperactivity disrupt intracellular copper transport through the suppression of Atox1 expression, ultimately resulting in endothelial copper overload.

### Superior cervical ganglionectomy effectively improved cardiac microvascular endothelial dysfunction

**Fig. 6.**
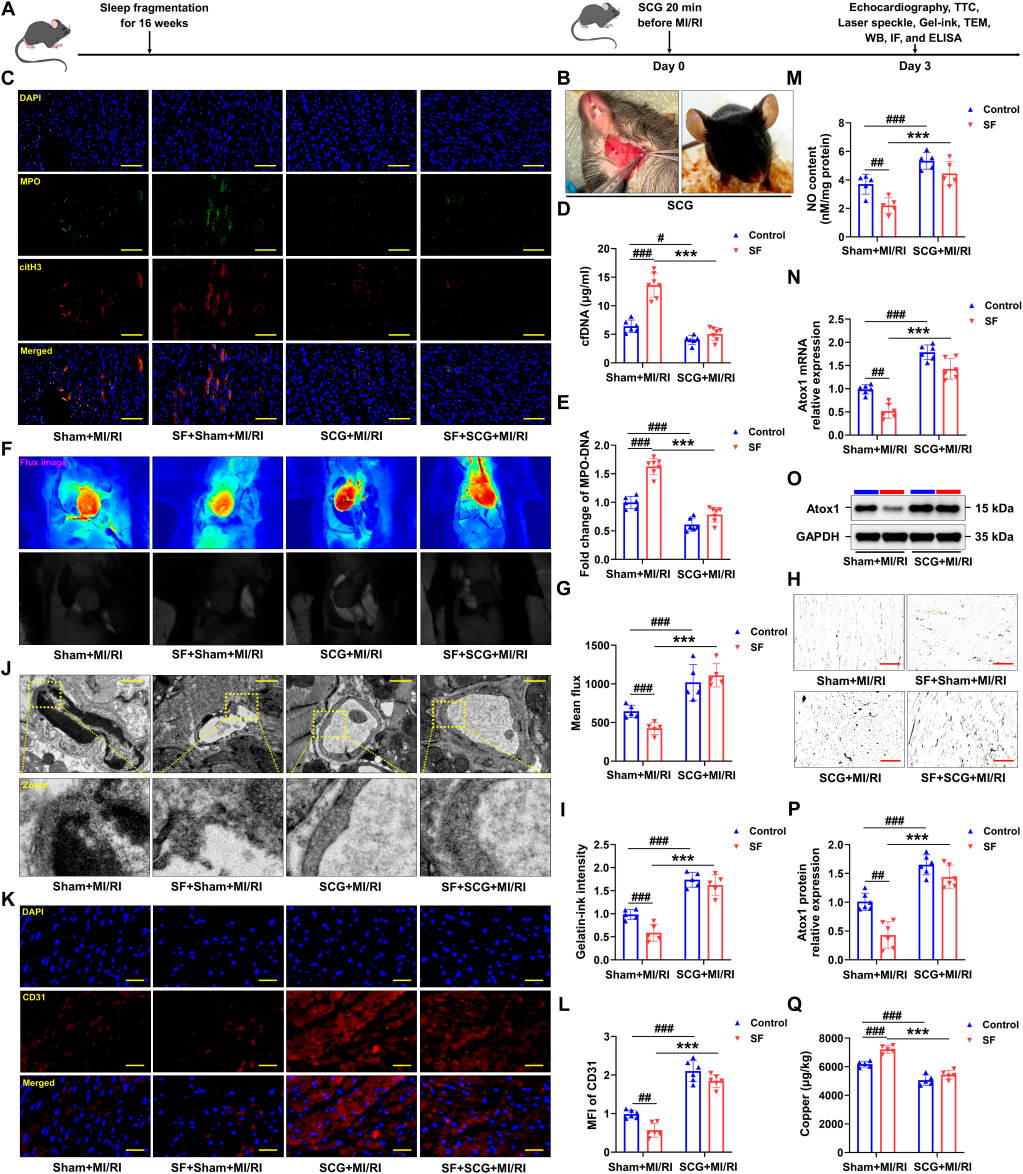
SCG reduced NET formation and attenuated cardiac microvascular injury in mice with SF. (A) Schematic of the experimental protocol used to establish SCG-attenuated MI/RI. (B) Removal of the ganglion from the posterior aspect of the common carotid artery was performed under a stereomicroscope, and images of mice that developed ptosis after the ganglion removed from one side were captured. (C) Representative images of immunofluorescence staining of NETs in myocardial tissues from Sham + MI/RI, SF + Sham + MI/RI, SCG + MI/RI, and SF + SCG + MI/RI groups (green: MPO, red: citH3, blue: DAPI) (*n* = 6). (D) Statistical analysis of cfDNA (*n* = 6 and 7 mice for Sham + MI/RI and SF + Sham + MI/RI group, *n* = 6 and 7 mice for SCG + MI/RI and SF + SCG + MI/RI group). (E) The MPO–DNA complex levels were assessed in SCG-treated mice (*n* = 6 and 7 mice for Sham + MI/RI and SF + Sham + MI/RI group, *n* = 6 and 7 mice for SCG + MI/RI and SF + SCG + MI/RI group). (F) The LASCA technique was used to assess the flow velocity in the infarct area of various groups. (G) The mean flux was qualified (*n* = 5 per group). (H and I) After MI/RI, hearts were injected with gelatin–ink, and samples were observed via microscope (*n* = 5 per group). (J) Electron microscope was used to observe the ultrastructural alterations of cardiac microvessels in mice treated by SCG (*n* = 5 per group). (K and L) Fluorescence imaging of CD31 in myocardial tissue of MI/RI-induced mice with SF treated by SCG (*n* = 6 per group). (M) Statistical analysis of NO content in myocardial tissues from each group (*n* = 5 per group). (N) The mRNA levels of Atox1 in each group were measured by qRT-PCR (*n* = 6 per group). (O and P) The protein level of Atox1 was analyzed by Western blotting (*n* = 6 per group). (Q) Copper ions in myocardial tissue of mice with SF were detected (*n* = 5 per group). Data are presented as mean ± SD. ^#^*P* < 0.05 versus Sham + MI/RI group; ^##^*P* < 0.01 versus Sham + MI/RI group; ^###^*P* < 0.001 versus Sham + MI/RI group; ^***^P < 0.001 versus SF + Sham + MI/RI group. Scale bars, 50 μm (C), 1 μm (J), and 25 μm (K).

In vivo, it was proposed that the heightened sympathetic activity observed in mice subjected to SF might be attributed to intensified signal transmission originating from the superior cervical ganglion. Consequently, superior cervical ganglionectomy (SCG) was conducted 20 min prior to MI/RI induction (Fig. 6A and B). The procedure was found to markedly diminish NET formation (Fig. 6C to E), attenuate MI/RI severity (Fig. S7A to E), and facilitate the restoration of blood flow (Fig. 6F and G) in SF-treated mice. Additionally, SCG ameliorated microvascular loss, as evidenced by gelatin–ink staining (Fig. 6H and I), reversed irregular microvascular walls, as observed via EM (Fig. 6J), and mitigated endothelial injury, as determined by immunofluorescence and NO analyses (Fig. 6K to M). Mechanistically, SCG reinstated Atox1 expression levels in SF mice to those comparable with control mice (Fig. 6N to P). Moreover, copper level differences (Fig. 6Q) were observed between SF-treated mice and control mice following MI/RI were all alleviated by SCG. Collectively, sympathetic hyperactivity is demonstrated to be a significant mechanistic contributor linking SF with the aggravation of cardiac microvascular endothelial injury.

### Vagal ES inhibited MI/RI in mice with SF by reducing copper overload

**Fig. 7.**
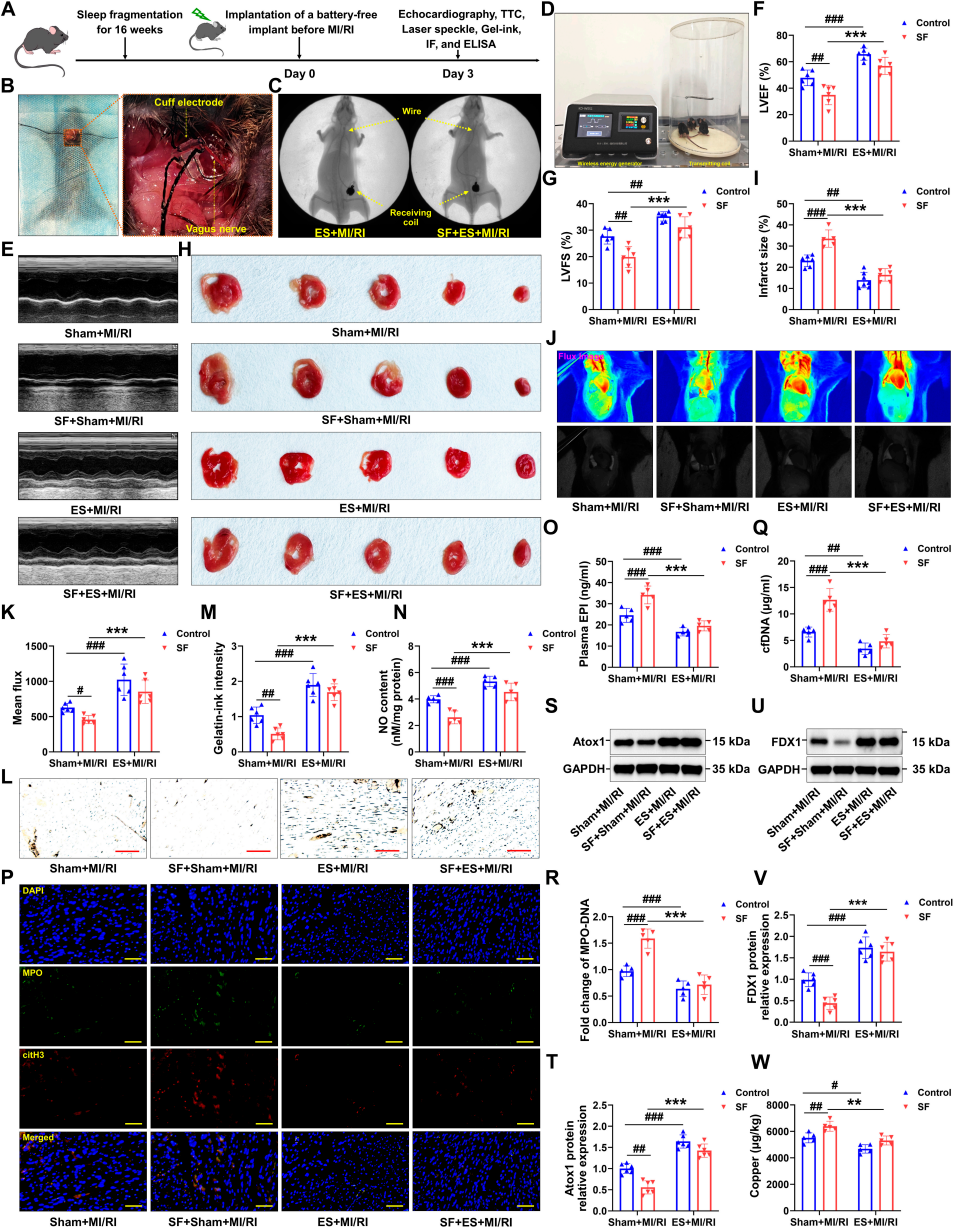
Vagal ES suppressed MI/RI in mice with SF by reducing copper overload. (A) Schematic of the experimental protocol used to establish vagal ES-suppressed MI/RI. (B) Schematic diagram of cuff electrode implantation on the surface of vagus nerve. (C) The x-ray images of the ES groups were observed. (D) Physical picture of the ES device. (E) Representative M-mode echocardiographic changes. (F and G) LVEF and LVFS were assessed by echocardiography (*n* = 6 per group). (H and I) Detection of infarct areas in cardiac tissue sections with TTC staining after treatment with ES (*n* = 6 and 5 mice for Sham + MI/RI and SF + Sham + MI/RI group, *n* = 7 and 6 mice for ES + MI/RI and SF + ES + MI/RI group). (J and K) The LASCA technique was used to assess the flow velocity in the infarct area of various groups (*n* = 6 per group). (L and M) After ES, hearts were injected with gelatin–ink, and samples were observed via microscope (*n* = 6 per group). (N) Statistical analysis of NO content in myocardial tissues (*n* = 5 per group). (O) ELISA was performed to detect EPI in the plasma (*n* = 5 per group). (P) Representative images of immunofluorescence staining of NETs in myocardial tissues from each group (green: MPO, red: citH3, blue: DAPI) (*n* = 6 per group). (Q) cfDNA in plasma in mice of each group (*n* = 5 per group). (R) The MPO–DNA complex levels were assessed in ES-treated mice (*n* = 5 per group). (S and T) The protein level of Atox1 was analyzed by Western blotting (*n* = 6 per group). (U and V) Validation of the levels of iron–sulfur cluster proteins by Western blotting after treatment with ES (*n* = 6 per group). (W) Copper ions in hearts of mice were detected (*n* = 5 per group). Data are presented as mean ± SD. ^#^*P* < 0.05 versus Sham + MI/RI group; ^##^*P* < 0.01 versus Sham + MI/RI group; ^###^*P* < 0.001 versus Sham + MI/RI group; ^**^P < 0.01 versus SF + Sham + MI/RI group; ^***^P < 0.001 versus SF + Sham + MI/RI group. ES, electrical stimulation. Scale bars, 50 μm.

Preclinical and clinical studies have consistently demonstrated the therapeutic efficacy of vagus nerve stimulation in a variety of cardiovascular and cerebrovascular conditions, including cardiac arrest, arrhythmias, myocardial infarction, cardiac hypertrophy, heart failure, and stroke [48–51]. Activation of afferent fibers through bioelectric stimulation affects the central regulation of the sympathetic and parasympathetic nervous systems [52]. Therefore, the effects of direct vagal ES delivered by the battery-free implant on MI/RI were subsequently evaluated. The timeline of the study was depicted in Fig. 7A. Before I/R, the left vagus nerve of the mice was implanted with a cuff electrode. Specifically, the vagus nerve at the stimulation site was enclosed within a longitudinally slit envelope, which was securely sealed using multiple bioabsorbable sutures (Fig. 7B). Receiving coils were positioned intermuscularly, beneath the skin. Figure 7C provides a magnified x-ray view of the implanted site. A fully implantable, battery-free, wireless ES device was employed (Fig. 7D). Echocardiographic assessment and 2,3,5-triphenyltetrazolium chloride (TTC) staining revealed a substantial increase in left ventricular ejection fraction (LVEF) and left ventricular fractional shortening (LVFS), and a significant decrease in infarction area in mice treated with ES (10V), in contrast to the MI/RI or SF + MI/RI group (Fig. 7E to I). Additionally, ES facilitated the restoration of blood flow (Fig. 7J and K) in SF-treated mice. Vagal ES ameliorated microvascular loss, as evidenced by gelatin–ink staining (Fig. 7L and M). The detection of NO and EPI showed that ES markedly increased the production of NO and reduced the plasma EPI compared with the MI/RI or SF + MI/RI group (Fig. 7N and O). We also found that vagal ES significantly diminished NE chemotaxis (Fig. S8A) and NET formation (Fig. 7P to R), up-regulated the protein expression of Atox1 and FDX1 (Fig. 7S to V), and reduced the level of copper (Fig. 7W). These results suggest that direct vagal ES through the battery-free implant effectively suppresses SF-aggravated MI/RI by reducing endothelial copper overload.

### Preparation and characterization of CRPPR@TTM

**Fig. 8.**
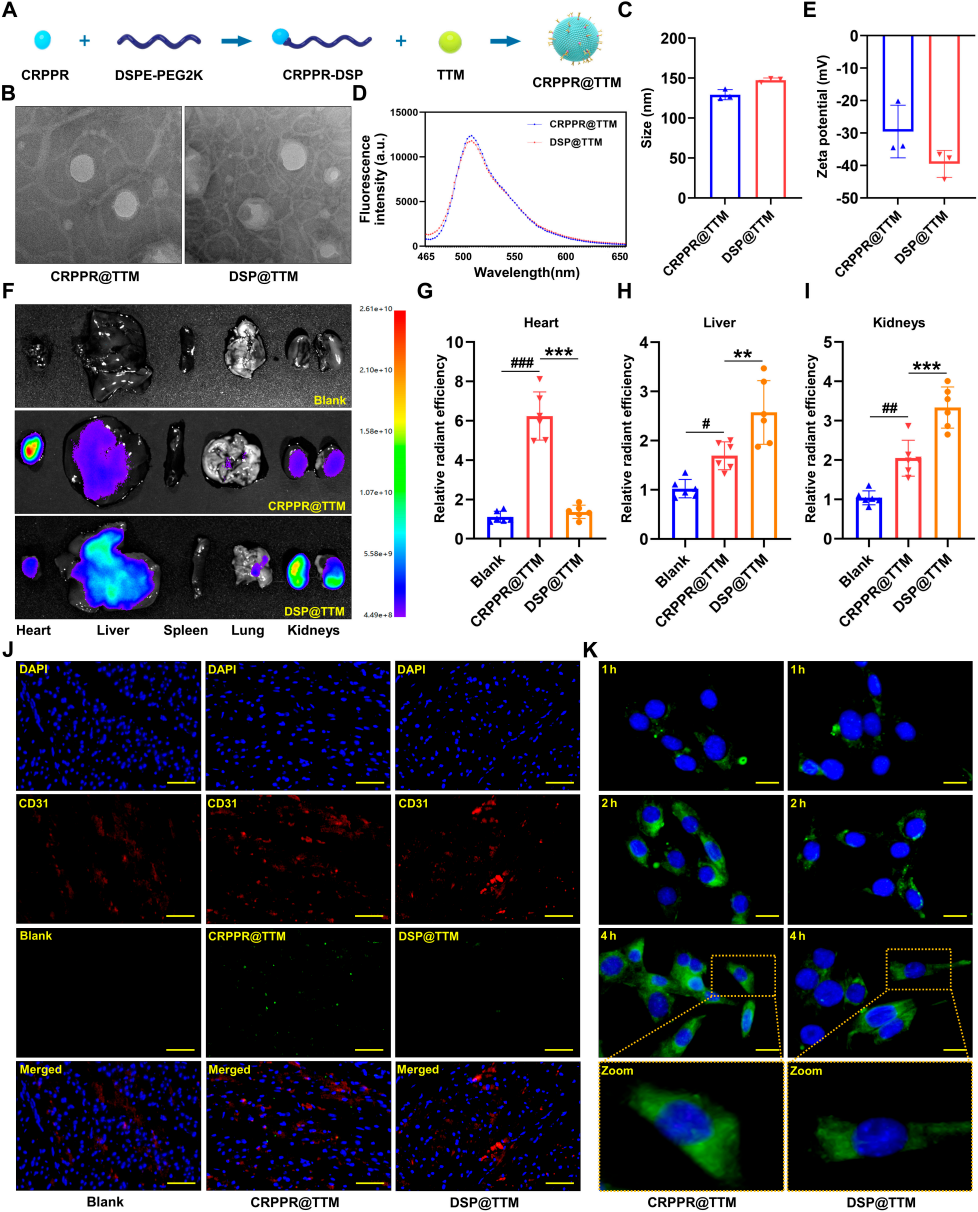
Preparation and characteristics of CRPPR@TTM. (A) Schematic illustration of CRPPR@TTM preparation. (B) Electron microscopic images of CRPPR@TTM and DSP@TTM (*n* = 3 per group). (C) Size of CRPPR@TTM and DSP@TTM (*n* = 3 per group). (D) Fluorescence spectrum analysis of CRPPR@TTM and DSP@TTM (*n* = 3 per group). (E) Surface charge of CRPPR@TTM and DSP@TTM determined using the zeta potential and dynamic light scattering (*n* = 3 per group). (F) Representative in vivo fluorescence in the heart, liver, spleen, lung, and kidneys at 2 h after administration with CRPPR@TTM or DSP@TTM (*n* = 6 per group). (G to I) Quantification of fluorescence intensity in the heart, liver, and kidneys (*n* = 6 per group). (J) Representative fluorescence images of CD31 (red), CRPPR@TTM (green), and DSP@TTM (green) in the heart of each group (*n* = 6 per group). (K) Immunofluorescence images showing intracellular localization of CRPPR@TTM or DSP@TTM within CMECs at different time points. Green-labeled CRPPR@TTM and DSP@TTM with DAPI-stained nuclei (blue) (*n* = 3 per group). Data are presented as mean ± SD. ^#^*P* < 0.05 versus Blank group; ^##^*P* < 0.01 versus Blank group; ^###^*P* < 0.001 versus Blank group; ^**^P < 0.01 versus CRPPR@TTM group; ^***^P < 0.001 versus CRPPR@TTM group. Scale bars, 50 μm (J) and 10 μm (K).

Ammonium tetrathiomolybdate (TTM), which has been widely employed to investigate the pathological impact of disrupted copper homeostasis in various disease contexts [53], was utilized to chelate copper ions. Additionally, DSPE-PEG2000 was modified to function as an efficient carrier for TTM (DSP@TTM), with a specific targeting mechanism toward CMECs. In vitro screening studies have previously identified that the short linear peptide CRPPR has a specific affinity for cardiac endothelium [54]. Based on this, the potential of CRPPR to selectively deliver TTM to cardiac endothelial cells was investigated. To this end, CRPPR was covalently conjugated to DSPE-PEG2000 to generate CRPPR@TTM for CMEC-targeted delivery. The schematic strategy for the synthesis of CRPPR@TTM is depicted in Fig. 8A. EM analysis revealed that CRPPR@TTM nanoparticles displayed uniform, spherical morphology with an approximate mean diameter of 130 nm (Fig. 8B and C). Furthermore, fluorescence spectral analysis indicated that both CRPPR@TTM and DSP@TTM (lacking CRPPR) exhibited strong fluorescence intensity (Fig. 8D). The zeta potentials of CRPPR@TTM and DSP@TTM, determined by dynamic laser scattering, were found to be −29.54 ± 9.37 mV and −39.51 ± 4.76 mV, respectively (Fig. 8E). Subsequently, the in vivo nanoparticle delivery efficiency was assessed. CRPPR@TTM and DSP@TTM nanoparticles were intravenously administered to mice, and 2 h post-injection, major organs (heart, liver, spleen, lung, and kidneys) were harvested for fluorescence intensity evaluation using in vivo imaging system (IVIS). Representative IVIS images demonstrated that the fluorescence intensity in the cardiac tissue was markedly greater in the CRPPR@TTM group compared to the DSP@TTM group. In contrast, elevated fluorescence signals were detected in the kidneys and liver of mice treated with DSP@TTM compared to CRPPR@TTM (Fig. 8F to I). Moreover, fluorescence microscope was employed to obtain fluorescence images of heart sections, enabling visualization of cellular internalization of CRPPR@TTM and DSP@TTM (green). CMECs within cardiac microvessels were labeled in red via immunofluorescent staining for CD31. Notably, the accumulation of CRPPR@TTM was markedly enriched in CMECs relative to DSP@TTM (Fig. 8J). In vitro imaging revealed that CMECs treated with CRPPR@TTM nanoparticles exhibited stronger green fluorescence at 1, 2, and 4 h than those treated with DSP@TTM nanospheres (Fig. 8K), suggesting that the presence of the CRPPR ligand substantially promoted nanocarrier uptake into CMECs. Collectively, these findings confirm that CRPPR@TTM effectively facilitates the targeted delivery of TTM into CMECs.

### Targeted therapy of CRPPR@TTM on SF-induced mice with MI/RI by inhibiting CMEC cuproptosis

**Fig. 9.**
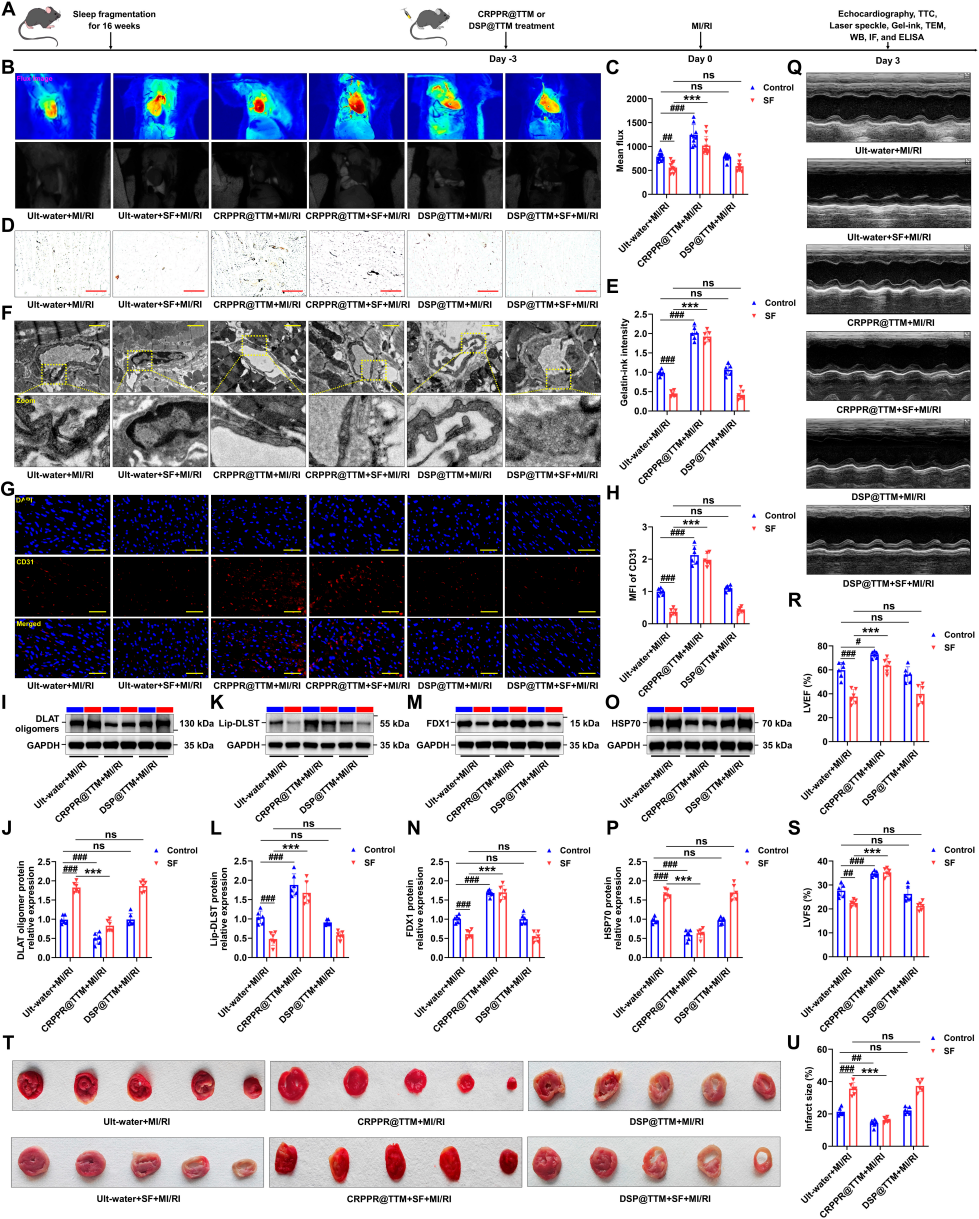
CRPPR@TTM attenuated cardiac microvascular injury and cuproptosis in mice with SF. (A) Schematic of the experimental protocol used to establish chronic SF-exacerbated MI/RI and treat with CRPPR@TTM or DSP@TTM for 6 d. (B) The LASCA technique was used to assess the flow velocity in the infarct area of various groups. (C) The mean flux was qualified (*n* = 11 and 10 mice for Ult-water + MI/RI and Ult-water + SF + MI/RI group, *n* = 9 and 10 mice for CRPPR@TTM + MI/RI and CRPPR@TTM + SF + MI/RI group, *n* = 9 mice for DSP@TTM + MI/RI and DSP@TTM + SF + MI/RI group). (D and E) After treatment with CRPPR@TTM or DSP@TTM, hearts were injected with gelatin–ink, and samples were observed via microscope (*n* = 6 per group). (F) Electron microscope was used to observe the ultrastructural alterations of cardiac microvessels in mice treated by CRPPR@TTM or DSP@TTM (*n* = 6 per group). (G and H) Fluorescence imaging of CD31 in myocardial tissue of MI/RI-induced mice with SF treated by CRPPR@TTM or DSP@TTM (*n* = 6 per group). (I to P) Validation of the levels of DLAT oligomers, lipoylated proteins, iron–sulfur cluster proteins, and HSP70 by Western blotting after treatment with CRPPR@TTM or DSP@TTM (*n* = 6 per group). (Q) Representative M-mode echocardiographic changes showed that CRPPR@TTM attenuated the impaired cardiac function in mice with SF and MI/RI. (R and S) Statistical analysis of LVEF and LVFS (*n* = 6 per group). (T) Representative TTC staining of the myocardium. (U) The percentage of infarct area was determined by TTC staining (*n* = 6 per group). Data are presented as mean ± SD. ^#^*P* < 0.05 versus Ult-water + MI/RI group; ^##^*P* < 0.01 versus Ult-water + MI/RI group; ^###^*P* < 0.001 versus Ult-water + MI/RI group; ^***^P < 0.001 versus Ult-water + SF + MI/RI group. Scale bars, 50 μm (D and G) and 1 μm (F). ns, not significant.

Firstly, the potential toxicity of CRPPR@TTM and DSP@TTM in normal mice was evaluated by assessing morphological alterations in the heart, liver, spleen, lung, and kidneys through hematoxylin and eosin (H&E) staining. The results demonstrated that neither CRPPR@TTM nor DSP@TTM exerted detectable toxic effects on these organs (Fig. S9A). Furthermore, the plasma levels of inflammatory cytokines, including IL-1β, IL-6, and IL-18, remained unchanged in normal mice following administration of CRPPR@TTM and DSP@TTM, respectively (Fig. S9B to D). Subsequently, in the experimental model depicted in Fig. 9A, pretreatment with CRPPR@TTM substantially enhanced blood flow restoration and mitigated microvascular loss (Fig. 9B to E). Ultrastructural changes in microvessels with or without CRPPR@TTM exposure were further visualized using EM. As presented in Fig. 9F, hearts from the SF + MI/RI group exhibited pronounced endothelial swelling and irregular microvascular walls in comparison to the MI/RI group. In contrast, structural impairment of the microvasculature induced by MI/RI was effectively reversed by CRPPR@TTM but not by DSP@TTM. Consistently, fluorescence-based histological staining revealed that only CRPPR@TTM markedly enhanced CD31 signal intensity (Fig. 9G and H). Moreover, CRPPR@TTM treatment inhibited DLAT oligomerization (Fig. 9I and J), restored lipoylated protein content (Fig. 9K and L), preserved iron–sulfur cluster proteins (Fig. 9M and N), and suppressed the elevation of HSP70 expression (Fig. 9O and P), thereby mitigating cuproptosis in SF mice subjected to MI/RI. To evaluate the antioxidant efficacy of CRPPR@TTM in MI/RI, ROS, SOD, GSH, and MDA levels were quantified in left ventricular tissues subjected to I/R. CRPPR@TTM treatment markedly suppressed the I/R-triggered increase in ROS and MDA. Furthermore, the CRPPR@TTM groups demonstrated significantly enhanced SOD activity and GSH content in injured myocardium compared to the MI/RI control (Fig. S10A to D). In addition, treatment with CRPPR@TTM led to a marked attenuation of MI/RI in SF mice (Fig. 9Q to U). Collectively, these results suggest that CRPPR@TTM ameliorates cardiac microvascular endothelial injury by suppressing cuproptosis in the context of SF-aggravated MI/RI.

### Evaluation of the efficacy of GSK against MI/RI mice with SF

**Fig. 10.**
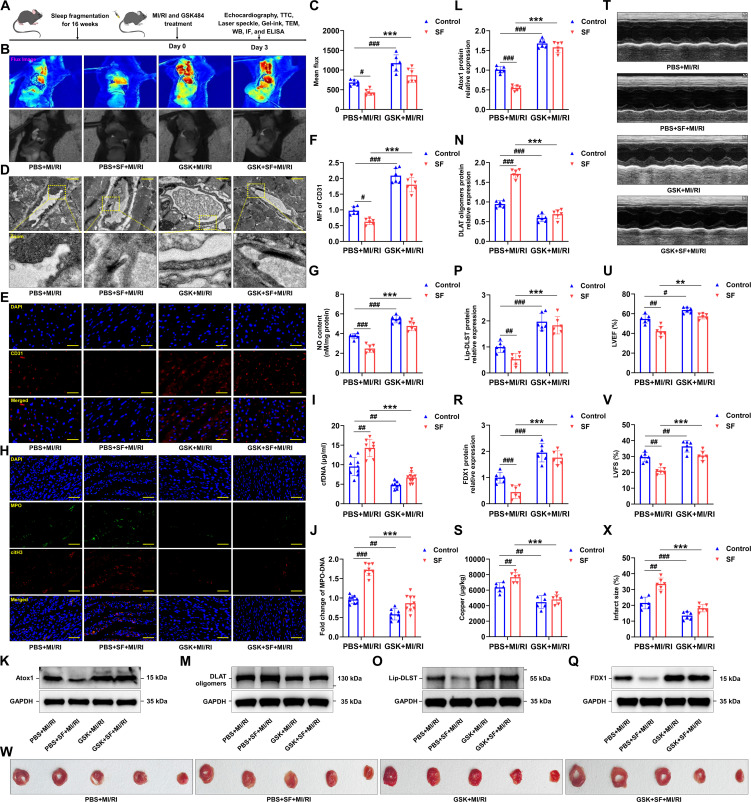
GSK diminished NET formation, cuproptosis, and cardiac microvascular injury in mice with SF. (A) Schematic of the experimental protocol used to establish chronic SF-exacerbated MI/RI and treat with GSK484 (GSK) for 3 d. (B) The LASCA technique was used to assess the flow velocity in the infarct area of various groups. (C) The mean flux was qualified (*n* = 6 per group). (D) Electron microscope was used to observe the ultrastructural alterations of cardiac microvessels in mice treated by GSK (*n* = 7 and 6 mice for PBS + MI/RI and PBS + SF + MI/RI group, *n* = 6 and 7 mice for GSK + MI/RI and GSK + SF + MI/RI group). (E and F) Fluorescence imaging of CD31 in myocardial tissue of MI/RI-induced mice with SF treated by GSK (*n* = 6 per group). (G) Statistical analysis of NO content in myocardial tissues from each group (*n* = 6 per group). (H) Representative images of immunofluorescence staining of NETs in myocardial tissues from each group (green: MPO, red: citH3, blue: DAPI) (*n* = 6 per group). (I) Detection of cfDNA in mice of each group (*n* = 9 and 8 mice for PBS + MI/RI and PBS + SF + MI/RI group, *n* = 8 and 10 mice for GSK + MI/RI and GSK + SF + MI/RI group). (J) The MPO–DNA complex levels were assessed (*n* = 9 and 8 mice for PBS + MI/RI and PBS + SF + MI/RI group, *n* = 8 and 10 mice for GSK + MI/RI and GSK + SF + MI/RI group). (K and L) The protein level of Atox1 was analyzed by Western blotting (*n* = 6 per group). (M to R) Validation of the levels of DLAT oligomers, lipoylated proteins, and iron–sulfur cluster proteins by Western blotting after treatment with GSK (*n* = 6 per group). (S) Copper ions in hearts of mice were detected (*n* = 6 per group). (T) Representative M-mode echocardiographic changes showed that GSK attenuated the impaired cardiac function in mice with SF and MI/RI. (U and V) Statistical analysis of LVEF and LVFS (*n* = 6 per group). (W) Representative TTC staining of the myocardium. (X) The percentage of infarct area was determined by TTC staining (*n* = 6 per group). Data are presented as mean ± SD. ^#^*P* < 0.05 versus PBS + MI/RI group; ^##^*P* < 0.01 versus PBS + MI/RI group; ^###^*P* < 0.001 versus PBS + MI/RI group; ^**^P < 0.01 versus PBS + SF + MI/RI group; ^***^P < 0.001 versus PBS + SF + MI/RI group. Scale bars, 1 μm (D), 25 μm (E), and 50 μm (H).

Given that GSK484 (GSK), a selective PAD4 inhibitor with strong specificity over other PAD family enzymes [55], was recently reported to inhibit PAD4, a central mediator of NETosis, it was employed to further substantiate the role of NETs in SF-aggravated MI/RI (Fig. 10A). As anticipated, administration of GSK markedly enhanced blood flow recovery (Fig. 10B and C) and ameliorated the irregular morphology and luminal stenosis of cardiac microvessels (Fig. 10D). In parallel, results from fluorescence staining and NO quantification revealed that GSK treatment elevated CD31 fluorescence intensity and facilitated NO production (Fig. 10E to G). Moreover, a notable reduction in colabeled citH3 and MPO levels was observed in the GSK-treated group relative to phosphate-buffered saline (PBS) controls (Fig. 10H). The formation of NETs in SF + MI/RI mice was also diminished, as evidenced by decreased cfDNA and MPO–DNA complexes following GSK intervention (Fig. 10I and J). Additionally, treatment with GSK up-regulated Atox1 expression (Fig. 10K and L), suppressed DLAT oligomerization (Fig. 10M and N), restored lipoylated protein abundance (Fig. 10O and P), preserved iron–sulfur cluster proteins (Fig. 10Q and R), and reduced the level of copper (Fig. 10S). The echocardiographic and TTC staining analyses demonstrated that GSK markedly enhanced cardiac function while substantially reducing myocardial infarction area in SF mice with MI/RI (Fig. 10T to X).‌ Collectively, GSK administration markedly suppresses NET formation and subsequently inhibits CMEC cuproptosis, supporting a causal relationship between NETs and the aggravation of cardiac microvascular damage in MI/RI mice under SF conditions.

## Discussion

**Fig. 11.**
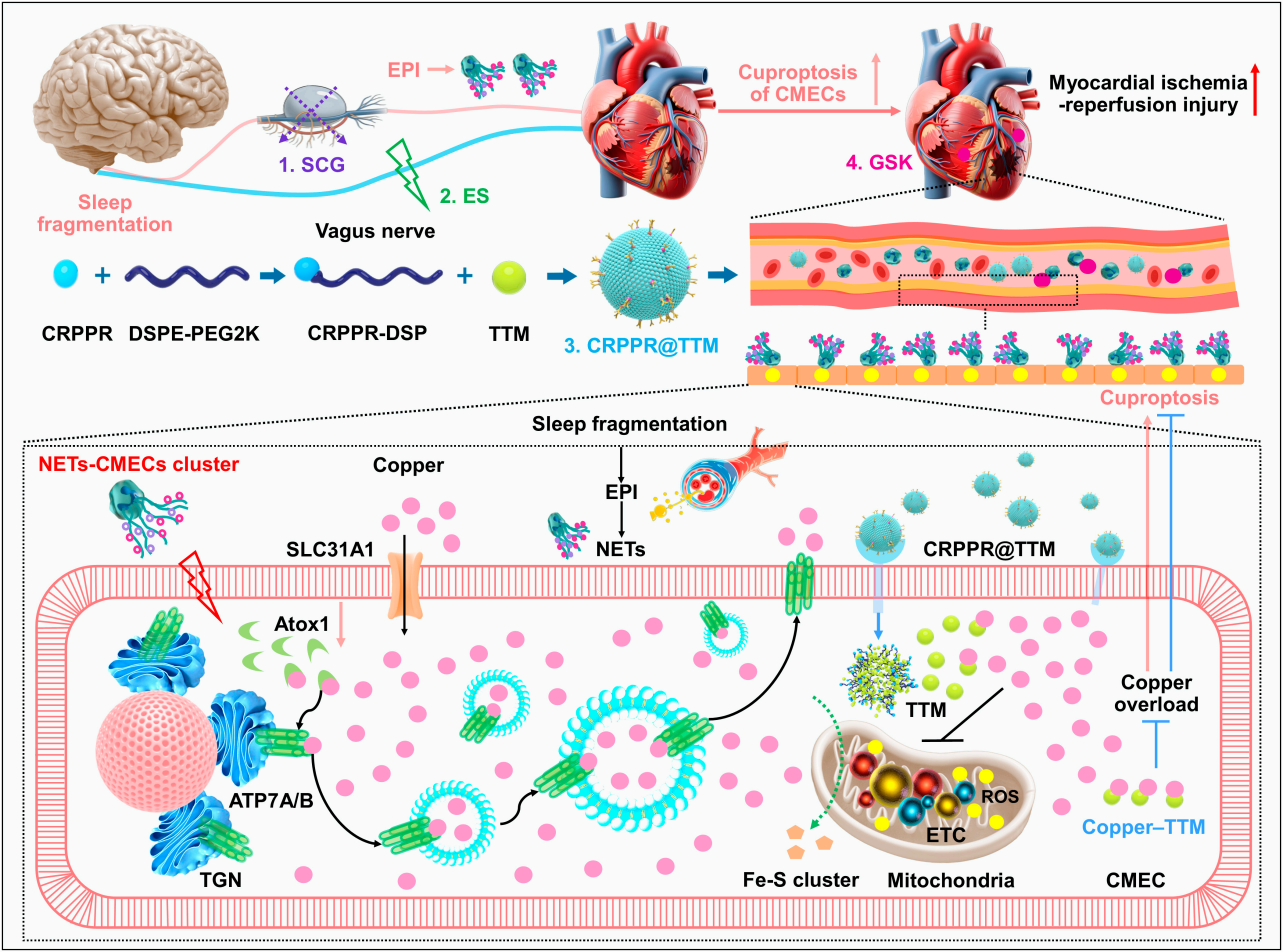
Schematic illustration of SF exacerbating MI/RI by inducing CMEC copper overload. SF-induced sympathetic hyperactivation facilitated NET formation. Then, NETs suppressed Atox1 expression, impaired ATP7A-mediated copper transport, and contributed to copper accumulation within CMECs. This copper overload further augmented cuproptosis and oxidative stress, while these pathological alterations were shown to be reversible through (1) sympathetic denervation, (2) vagal ES, (3) targeted delivery of copper chelators, or (4) the inhibition of NETs.

Sleep disorders have been associated with elevated mortality rate of cardiovascular diseases, and the objective of the present research was to explore the brain–heart regulatory mechanisms involved in sleep disturbance. The findings indicated that SF induced sympathetic hyperactivation, elevated EPI levels, and facilitated the chemotactic migration of NEs toward cardiac tissue, subsequently promoting NET formation. This process suppressed Atox1 expression, disrupted intracellular copper trafficking, and resulted in copper accumulation within CMECs. The consequent copper overload was found to aggravate cuproptosis and contribute to cardiac microvascular endothelial dysfunction following MI/RI. Notably, these pathological outcomes were ameliorated through SCG, treatment with copper chelators, or administration of GSK (Fig. 11). The study provides mechanistic insight into how sleep disorders intensify myocardial injury and identifies potential therapeutic strategies for intervention.

Although short-term sleep deprivation has been associated with diminished sympathetic activity [56], prolonged deprivation is known to induce sustained hyperactivation of the sympathetic system [57]. The latter observation corresponds with the current findings derived from 16 weeks of fragmented sleep. Cardiac sympathetic hyperactivity has been implicated in the elevated risk of hypertension [58], arrhythmogenesis [59], endothelial dysfunction [60], and myocardial metabolic disturbances [61]. Moreover, the present investigation demonstrated that heightened sympathetic tone contributed to copper metabolic dysregulation in CMECs, further aggravating cuproptosis and cardiac microvascular endothelial impairment following MI/RI. Prior functional investigations have demonstrated that sleep deprivation enhances sympathetic nerve activity, consistent with the current findings and likely due to central nervous system-mediated modulation of superior cervical ganglion activity [62]. This observation not only offers additional anatomical validation of brain–heart communication but also posits that sleep disorder-induced sympathetic overactivity involves not only functional abnormalities but also structural remodeling, thereby highlighting the therapeutic relevance of modulating sympathetic excitability in the management of insomnia.

During the process of NETosis, NEs have been shown to release NETs that facilitate platelet aggregation and promote thrombus development, thereby exacerbating inflammation and triggering innate immune activation, which may ultimately result in cardiac injury [63]. Nevertheless, the contribution of NEs to myocardial damage appears to be multifactorial. Certain investigations have reported that pharmacological inhibition of NEs alleviates acute inflammation in murine models of viral myocarditis [64]. Additionally, previous findings have revealed that NE depletion reduces infarct size in mouse models of MI/RI, which aligns with the current observations. However, the link between SF and inflammatory response during MI/RI remains incompletely elucidated. To assess myocardial damage and its related inflammatory cascade, concentrations of IL-1β, IL-6, and IL-18 were measured following MI/RI in mice subjected to SF pretreatment. The results demonstrated that the levels of IL-1β, IL-6, and IL-18 were markedly elevated in MI/RI animals exposed to SF. Subsequently, NET release in cardiac tissues was examined to investigate the involvement of NETs in SF after MI/RI. Compared with MI/RI-only mice, SF-treated counterparts exhibited pronounced inflammatory cell infiltration, along with increased levels of citH3, cfDNA, and MPO–DNA, suggesting a potential association between NETosis and SF. Collectively, these murine data indicate that NET accumulation driven by SF serves a pivotal function in the pathogenesis of MI/RI and is positively correlated with disease severity.

The disruption of metal ion homeostasis has been identified as a pivotal factor in the pathogenesis of MI/RI [65]. Previous research indicated that copper ion concentrations rise rapidly within myocardial tissue during MI/RI episodes [66]. Excess copper has been shown to elevate intracellular oxidative stress, initiate apoptotic signaling cascades, interfere with mitochondrial respiratory chain function, and induce cuproptosis. Prolonged exposure to copper overload has also been reported to impair myocardial contractile performance by suppressing calcium uptake in the sarcoplasmic reticulum [67]. A body of evidence has suggested an association between dysregulated copper metabolism and organ damage [68]. In line with this, the present study confirmed the occurrence of myocardial cuproptosis under MI/RI conditions. Additionally, copper overload was demonstrated to act as a central mediator linking sympathetic hyperactivity to the exacerbation of cardiac microvascular endothelial injury in SF-exposed MI/RI mice. Notably, the targeted delivery of TTM to CMECs not only mitigated the aggravating influence of SF on MI/RI but also alleviated myocardial injury directly caused by MI/RI, thereby proposing a potential therapeutic approach for MI/RI attenuation.

EPI serves as the principal neurotransmitter released at sympathetic nerve terminals. In the current investigation, EPI was observed to markedly enhance NET formation under in vitro conditions. Moreover, we successfully established a NET-induced copper overload model in CMECs. In parallel, in vivo sympathetic ganglionectomy in SF-exposed mice reduced NET formation, alleviated endothelial copper accumulation, and mitigated cuproptosis following MI/RI. These results emphasize the pivotal regulatory role of sympathetic nerve signaling in modulating intracellular copper metabolism. Given the ability of sympathetic pathways to modulate copper levels, the molecular mechanism underlying sympathetic signal regulation of copper transport in CMECs was further investigated. According to prior studies [69], the copper transporter ATP7A facilitates the export of copper from the trans-Golgi network (TGN) via the formation of copper-laden vesicles—constituting the initial phase of copper efflux. Subsequently, ATP7A is mobilized toward the cell periphery, where vesicle fusion with the plasma membrane releases copper into the extracellular space. Thereafter, ATP7A undergoes endocytic retrieval, forming endosomes and returning to the TGN with assistance from the retromer complex. A complete copper efflux cycle necessitates dynamic recycling of ATP7A between the TGN and the plasma membrane. Therefore, ATP7A was utilized in this study as a critical tracer protein, and the copper-specific probe CS1 was employed to elucidate the intracellular copper accumulation mechanism driven by NETs. Intracellular copper trafficking constitutes a coordinated, multi-protein biological process. Atox1 functions by transferring intracellular copper to ATP7A, enabling the generation of copper-transporting vesicles [70]. It binds free cytosolic copper ions and delivers them specifically to ATP7A and ATP7B, ensuring efficient metal loading of these efflux pumps. This directed transfer mechanism facilitates copper export from the cell or bile excretion for systemic elimination. Thus, Atox1 operates as an essential upstream regulator of copper efflux, supporting the function of the core export machinery. In both the cardiac tissue of SF mice and NET-stimulated CMECs, a marked down-regulation of Atox1 protein expression was observed, which disrupted the copper trafficking pathway and led to cuproptosis.

Recent clinical evidence, as highlighted in translational studies, indeed demonstrates that serum markers associated with caspase-mediated apoptosis (e.g., p17 peptide) correlate with conventional necrosis markers (cTnI and CK-MB) in ST-elevation myocardial infarction patients. However, the correlation is not absolute, suggesting that both apoptosis and necrosis are involved in determining infarct size [71]. Our study did not quantify apoptosis directly; rather, our proteomic and functional data point to a shift in cell death modalities induced by chronic SF. While myocardial apoptosis undoubtedly occurs, the significance of apoptosis in cardiac myocytes remains a subject of investigation. Notably, experimental data from preclinical models challenge the dominant pathological role of caspase-dependent apoptosis in infarct size. Under the stress of SF and subsequent NET-mediated endothelial damage, the pathway leading to CMEC demise was not classical apoptosis but cuproptosis—a copper-dependent, caspase-independent form of death characterized by DLAT oligomerization, lipoylated protein loss, and HSP70 elevation. Our findings of increased DLAT oligomers and decreased FDX1 support the activation of this distinct pathway.

Thus, our study expands the paradigm of cell death in MI/RI by introducing cuproptosis as a critical player in ‌cardiac microvascular endothelium‌, likely operating in parallel or in synergy with other death pathways in cardiomyocytes. In this context, endothelial cuproptosis may contribute to microvascular dysfunction, a key determinant of “no-reflow” and final infarct expansion, rather than directly causing mass myocyte death. Targeting NET-triggered cuproptosis, as we demonstrated with CRPPR@TTM and GSK, may therefore salvage microvascular integrity, indirectly limiting infarct size by preserving the microcirculation—an effect potentially additive to strategies targeting cardiomyocyte apoptosis or necrosis. Future studies should directly compare the spatiotemporal dynamics and quantitative contributions of apoptosis, necrosis, ferroptosis, and cuproptosis in SF-aggravated MI/RI to fully elucidate their interplay.

The primary limitations of this study are outlined as follows: First, the experimental design was restricted to male mice. Although biological sex has been recognized as a factor influencing endothelial injury under sleep disorder conditions, the objective of this investigation centered on elucidating the regulatory role of the sympathetic nervous system in copper metabolism. Thus, future research is warranted to explore sex-specific differences in endothelial injury associated with sleep disorders in both male and female mice. Second, while copper overload has been identified as a pivotal mediator in sleep disorder-induced endothelial damage, the current study did not assess copper transport dysfunction in the myocardial tissue of patients with sleep disorders due to ethical limitations. This underscores the necessity for future development of noninvasive methodologies to address this gap. Third, although partial evidence was provided linking Atox1 suppression to copper metabolic disturbances, in vivo Atox1 knockout models were not employed to definitively establish the causal relationship between Atox1 inhibition, copper accumulation, and upstream mechanisms involved in brain–heart comorbidities. Lastly, although the results preliminarily suggest a direct involvement of copper overload in the progression of post-MI/RI pathology, the roles of Atox1 inhibition and copper accumulation in ischemic cardiomyopathy were not further delineated through in vivo validation, which remains a key focus for future investigation.

## Conclusion

In conclusion, a brain–heart communication mechanism was characterized, in which SF-induced sympathetic hyperactivation facilitates NET formation, subsequently leading to copper accumulation in CMECs and aggravating endothelial cuproptosis and oxidative stress following MI/RI. The findings identified copper metabolism as a sensing system within the cardiac microvascular endothelium, receiving input from central and sympathetic pathways and functioning as a regulatory node that governs downstream cuproptosis. Therefore, signaling pathways involved in endothelial copper transport may represent viable therapeutic targets for MI/RI associated with sleep disturbances.

## Materials and Methods

### Animals

Male C57BL/6 mice (aged 6 to 8 weeks, body mass 21 to 23 g) were acquired from the Experimental Animal Center of Harbin Medical University, Harbin, China. The animals were kept in standard plastic enclosures under regulated environmental parameters (temperature: 20 to 26 °C; humidity: 50% to 60%) with alternating 12-h light/dark periods, receiving unrestricted food and water access. The experimental procedures received approval from the Ethics Committees of Harbin Medical University. Randomization and blinding protocols were implemented. In brief, animals were randomly allocated to control and experimental cohorts following identification using ear tags. Echocardiographic assessments and cardiac I/R injury modeling were conducted by a trained operator blinded to group allocation. At all stages of the study, the individual responsible for procedural execution and data interpretation remained unaware of group designation.

### Preparation of SF mouse model

Mice were housed in a sleep deprivation apparatus (Anhui Yaokun Biotechnology, China, ZL-014). A sweep bar was programmed to traverse the cage floor at 2-min intervals during the light phase (ZT0 to ZT12) and remained immobile throughout the dark phase (ZT12 to ZT24) for 16 weeks. In contrast, control mice allowed to maintain normal sleep patterns were placed in identical chambers where the sweep bars remained stationary.

### Animal anesthesia

When required, all mice were anesthetized via inhalation of 4% to 6% (v/v) isoflurane (Shandong Ante Livestock Technology Co. Ltd., China), with maintenance anesthesia administered at a concentration of 2% (v/v) isoflurane. In procedures necessitating respiratory interruption, encompassing MI/RI modeling, tracheal intubation was carried out using a 20-gauge catheter. Mechanical ventilation was subsequently implemented through a rodent ventilator (Chengdu Taimeng Software Co. Ltd., China, HX-101E) set to an inspiratory-to-expiratory ratio of 1:1.5 and a respiratory rate of 120 breaths/min.

### Establishment of MI/RI model

To create the MI/RI experimental model, a ligation procedure was executed on the left anterior descending (LAD) coronary artery in mice, utilizing an established methodology. In brief, the animals were anesthetized, intubated, and placed on mechanical ventilation. The heart was exposed through a left-sided minithoracotomy performed between the fourth and fifth ribs. A 10-0 prolene suture (NINGBO MEDICAL, LingQiao) was positioned beneath the LAD, approximately 2 to 3 mm from its origin, between the left auricle and conus arteriosus. Complete LAD occlusion was achieved by securing the suture loop. The effective induction of localized ischemia was validated by observing S–T segment elevation in the electrocardiogram and noting the color alterations in the distal myocardial tissue. Following a 45-min ischemic period, reperfusion commenced upon release of the ligature, with visual confirmation of restoration. During the reperfusion phase, the animals recovered on heating pads and were maintained in enclosures with unlimited access to softened food and water. Control animals in the Sham group received identical surgical exposure but without LAD artery ligation. In the subsequent experiments, the mice were divided into the sham operation group, the model group, and the intervention group using the random number table method. Mice were included if they met the following: (a) successful I/R modeling confirmed by ST-segment elevation (>0.1 mV) and (b) no preexisting cardiac abnormalities. Mice were excluded if they died during surgery, failed to develop infarction, or showed severe post-operative complications.

### Implantation of a battery-free implant

A battery-free ES implant was designed and evaluated in-house. Prior to MI/RI preparation, the battery-free implant was implanted in the mice. Then, mice were performed by I/R, with the battery-free device for ES (10V), and stimulated for 30 min daily. In this study, the mice were subjected to MI/RI and treated with the above interventions randomly. Efficacy was evaluated based on morphology, transthoracic echocardiography, and biomarker analyses in a blinded fashion.

### Nanoparticle preparation and characterization

DSPE-PEG2K, cholesterol, soy lecithin, DSPE-PEG2K-CRPPR (Xi’an Ruixi Biological Technology Co. Ltd. China), and TTM (15060-55-6, purity 99.9%, MedChemExpress) were codissolved in 6 ml of chloroform, followed by evaporation to form a thin lipid film. Subsequently, nanoparticles were harvested by employing ultrasonication in conjunction with a liposome extruder. The nanotransfer device, equipped with a polycarbonate membrane (pore size 30 nm), ‌was used‌ for dialysis to remove unbound drugs. The morphological characteristics and particle dimensions were assessed via EM, while zeta potential and size distribution were determined using a dynamic light scattering detector (Zetasizer-ZS90). The intracellular uptake of CRPPR@TTM and DSP@TTM by CMECs was observed through fluorescence microscopy.

### Nanoparticle treatment

Following 16 weeks of either SF intervention or standard conditions, the SF and control mice were arbitrarily split into 3 distinct groups, respectively. Among them, mice received daily intravenous administrations of vehicle solution, CRPPR@TTM, or DSP@TTM (TTM at a dosage of 20 mg/kg) for 3 consecutive days prior to the induction of I/R until sacrificed animals. Efficacy was evaluated based on morphology, transthoracic echocardiography, and biomarker analyses in a blinded fashion.

### GSK treatment

Following 16 weeks with or without SF intervention, the SF and control mice were arbitrarily split into 2 separate groups, respectively. Among them, mice in the GSK group were administered GSK (1652591-81-5, purity 99.48%, MedChemExpress) at a dose of 4 mg/kg via daily intravenous injection for 3 consecutive days. The remaining group received an intravenous injection of the corresponding vehicle solution.

### TTC staining

We used TTC staining to assess whether sleep deprivation aggravated myocardial damage. To evaluate the infarct size, hearts were harvested 3 d following I/R. After excision and thorough rinsing in PBS to remove residual blood, the tissue was frozen at −20 °C for 15 min and subsequently sectioned transversely into 5 slices per heart, with each slice cut to a thickness of 1 to 2 mm. These sections were then incubated with TTC (T8877, Sigma) at 37 °C for 20 min, followed by fixation in 4% paraformaldehyde (PFA) for 24 h to demarcate the infarcted regions. Infarcted myocardium was expected to remain unstained, whereas viable myocardial tissue appeared red. Quantification of left ventricular infarct size was performed using ImageJ software and presented as the mean percentage of infarcted area relative to the total ventricular area.

### Detection of EM

Cardiac tissue specimens measuring 1 mm × 1 mm × 1 mm underwent fixation using 2.5% glutaraldehyde and subsequent treatment with 1% osmium tetroxide before ultrathin sectioning, and the identical fixation protocol was applied to cell samples designated for EM observation. The ultrastructure of cardiac microvessels and the presence of NETs were subsequently examined utilizing a Tecnai G2 transmission electron microscope (FEI, USA).

### LASCA of infarcted tissues

Assessment of the infarcted region’s flow velocity was conducted using a Moor FLPI-2 real-time blood flow zoom laser speckle imaging system (Moor Instruments Ltd., UK) for Lasca evaluation.

### Gelatin–ink staining

To examine microvascular patency, firstly, the mice were anesthetized and a gelatin–ink mixture (3% gelatin and ink) was administered into the heart through the jugular vein at room temperature (30 °C). The chest cavity was then opened, and the blood vessels connected to the heart were clamped using hemostatic forceps to prevent the gel ink from leaking out through the cardiac vessels. The tissue was subsequently incubated at 4 °C for 1 h to ensure complete solidification of the gel ink. Finally, frozen sections of the heart were prepared to observe the microvascular patency using light microscopy.

### Liquid chromatography–tandem mass spectrometry-based proteomic analysis

Proteins were procured from tissue specimens utilizing SDT lysis buffer (4% SDS, 100 mM dithiothreitol, 100 mM tris–HCl, pH 8.0). Digestion of proteins was conducted using the filter-aided sample preparation (FASP) method as described by Wiśniewski et al. [72]. Peptides from each sample were introduced into a 50-cm Low-Load μPAC Neo HPLC Column (Thermo Scientific) at a flow rate of 2.2 μl/min. MS data were processed using DIA-NN 1.8.1 and searched against the UniProtKB database. Bioinformatic analyses were performed in Microsoft Excel and the R statistical computing environment. Hierarchical clustering and volcano plot generation were conducted using the R language. For sequence annotation, data were retrieved from UniProtKB/Swiss-Prot, the Kyoto Encyclopedia of Genes and Genomes (KEGG), and GO. GO and KEGG enrichment analyses were performed using Fisher’s exact test, with false discovery rate (FDR) correction applied for multiple hypothesis testing. GO terms were categorized into 3 groups: biological process, molecular function, and cellular component. GO and KEGG pathways were considered markedly enriched when Fisher’s exact test yielded a *P* value of <0.01. Construction of protein–protein interaction networks was accomplished utilizing the STRING database and visualized through Cytoscape software.

### Quantification of cfDNA and MPO–DNA complexes

cfDNA present was quantified utilizing the Quant-It PicoGreen dsDNA kit (Thermo Fisher) per the supplier’s protocol. MPO–DNA complexes in plasma and supernatants were identified using a capture enzyme-linked immunosorbent assay (ELISA) (ZK-5494, ZHEN KE SHENG WU), as previously reported and carried out per the supplier’s protocols [73].

### Inflammatory factor detection

Whole blood from mice was procured and maintained at room temperature in test tubes for 30 min. Plasma and supernatants were separated by centrifugation, rapidly frozen in liquid nitrogen, and subsequently preserved at −80 °C for downstream analysis. The plasma concentrations of IL-1β (EM0029, HUABIO), IL-18 (EM0034, HUABIO), and IL-6 (EM0004, HUABIO) were determined using ELISA kits per the supplier’s protocol.

### ROS, SOD, GSH, and MDA assay

ROS levels [labeled by 2ʹ,7ʹ-dichlorodihydrofluorescein diacetate‌ (DCFH-DA)] were assessed using a ROS detection kit (S0033S, Beyotime, China). Formalin-fixed paraffin-embedded (FFPE) sections were deparaffinized, rehydrated, and permeabilized with 0.1% Triton X-100 for 10 min at room temperature. Sections were then incubated with 10 μM DCFH-DA for 30 min at 37 °C in a humidified, dark chamber. Negative control sections were incubated with DCFH-DA plus 10 mM N-acetylcysteine (NAC). After incubation, sections were washed 3 times with Hanks’ balanced salt solution (5 min each wash) to remove unbound probe. This protocol ensured specific intracellular ROS detection with minimized artifacts from leakage, auto-oxidation, or nonspecific binding. Fluorescence images were captured under a microscope, and quantitative analysis of fluorescence intensity was performed using ImageJ software. Furthermore, the detection of ROS in the cells was carried out strictly in accordance with the instructions of the kit.

Total SOD activity was quantified using the WST-8-based assay kit (S0101M, Beyotime, China). In this method, xanthine oxidase generates superoxide anions, which react with WST-8 to form a water-soluble formazan dye. SOD inhibits this reaction by catalyzing the dismutation of superoxide anions, resulting in a negative correlation between SOD activity and formazan production. The absorbance of the formazan product at 450 nm was measured to calculate SOD enzymatic activity.

Total GSH levels were measured using a commercially available assay kit (BC1175, Solarbio, China). Myocardial tissue and CMECs were prepared following the manufacturer’s protocol. A standard curve was generated by plotting absorbance values against known concentrations of GSH standards. The GSH content in samples was determined by comparing their absorbance readings to the standard curve.

MDA levels of cardiac tissue or CMECs were quantified using an MDA assay kit (KTB1050, Abbkine, China) according to the manufacturer’s instructions. MDA reacts with thiobarbituric acid to produce a brownish red trimethadione complex, which exhibits peak absorbance at 532 nm. Colorimetric analysis at this wavelength allowed estimation of MDA content. To minimize interference from sucrose, absorbance at 600 nm was simultaneously measured. The final MDA concentration was calculated by subtracting the 600-nm absorbance from the 532-nm value.

### Cell culture

CMECs were isolated from mice via trypsin and collagenase according to a previous study. All cells were kept at 37 °C in a humidified incubator comprising 5% CO₂ and 95% air. For subsequent treatments, NETs and CuCl₂ (Sigma, C3279) were separately dissolved and subsequently diluted with culture medium.

### Cell coculture

NEs were isolated and purified from mice according to previously reported methods [74]. Initially, CMECs were seeded into the lower compartment of the transwell chamber at a density of 5 × 10^4^ cells/cm^2^ in Dulbecco’s modified Eagle’s medium supplemented with 10% fetal bovine serum. The cells were then subjected to an H/R model, which involved 12 h of hypoxia (1% O₂, 94% N₂, and 5% CO₂) followed by 24 h of reoxygenation (21% O₂, 74% N₂, and 5% CO₂). Subsequently, the purified NEs were placed in the upper compartment of the transwell chamber, and 5 μM EPI was administered. After a 6-h incubation period, NE chemotaxis and NET formation were assessed.

### NET production and isolation

Briefly, NEs obtained from mice were suspended again in RPMI 1640 medium (Thermo Fisher) and transferred to 6-well plates with 1.8 × 10^6^ cells in each well. After exposure to 50 nM phorbol 12-myristate 13-acetate (PMA) for 4 h, the medium was extracted, and the wells underwent rinsing with RPMI 1640. This PMA concentration triggered NET formation characteristics without causing apoptotic or necrotic effects. Following supernatant removal, the attached NETs were separated through pipetting using 2 ml of ice-cold PBS and then underwent centrifugation at 1,000*g* for 10 min at 4 °C. The supernatant containing NETs was collected after centrifugation. These extracted NETs were utilized immediately or preserved through rapid freezing in liquid nitrogen.

### Copper detection

CS1 (Psaitong, C11621) [75], a designed fluorescent indicator applied in copper detection of viable cells, functions as a compact fluorochrome capable of penetrating membranes to detect mobile copper distributions across biological samples, particularly in active cellular systems. During the staining protocol, the growth medium was exchanged with 5 μM CS1, followed by a 20-min cell incubation period at 37 °C under dark conditions before fixation and additional staining steps. Nuclei were counterstained using 4′,6-diamidino-2-phenylindole (DAPI). CS1-associated fluorescence signals were captured via confocal laser scanning microscopy.

### Immunofluorescence staining

The tissue specimens underwent collection, fixation in 4% PFA, paraffin embedding, and sectioning to achieve 5-μm thickness. Antigen recovery utilized sodium citrate buffer (pH 6.0), preheated through microwave treatment for 5 min, succeeded by a 20-min solution immersion. Regarding cell cultures, PBS rinsing occurred 3 times, followed by fixation using 4% PFA containing 0.1% Triton X-100 for membrane penetration. The prepared specimens received blocking treatment with 1% bovine serum albumin (BSA) at ambient temperature for 60 min. The specimens were exposed to primary antibodies diluted in 1% BSA during overnight incubation at 4 °C. The subsequent day, after primary antibody elimination, corresponding secondary antibodies were introduced for 60 min at room temperature in dark conditions. The procedure concluded with DAPI nuclear staining, and image acquisition occurred via a laser confocal microscope.

### Western blot analysis

Myocardial tissues or cultured CMECs were lysed using radioimmunoprecipitation assay buffer. Equal amounts of protein (50 μg) were separated via sodium dodecyl sulfate–polyacrylamide gel electrophoresis, transferred onto a nitrocellulose membrane, and incubated overnight at 4 °C with specific primary antibodies, as listed in Table S1.

### qRT-PCR analysis

Total RNA was extracted from cardiac tissues and subsequently subjected to reverse transcription. Quantification of mRNA expression was conducted using quantitative reverse transcription polymerase chain reaction (qRT-PCR), with normalization to β-actin transcript levels. The primer sequences utilized in this study are depicted in Table S2.

### Plasma and myocardium EPI determination

EPI concentrations in both plasma and tissue homogenates were determined using an ELISA kit (Wuhan Huamei Biological Engineering, CSB-E08679m) per the supplier’s protocol.

### NO measurement‌

NO levels were measured by quantifying its stable metabolic end-products, nitrite (NO₂^−^) and nitrate (NO₃^−^), using a Griess reaction-based assay kit (Calbiochem). The assay detected the colored complex formed by reaction of NO₂^−^/NO₃^−^ with Griess reagents [sulfanilamide and N-(1-naphthyl) ethylenediamine] at 540 nm, with results expressed as nM/mg protein of NO equivalent.

### CMEC wound healing migration analysis

When the cells achieved 100% confluence, a sterile 200-μl pipette tip was employed to inflict a standardized straight-line wound at the center of the monolayer. The wound was then imaged using the phase-contrast microscopy function of an inverted microscope (Nikon, Tokyo, Japan). Subsequently, the cells were incubated under standard conditions for 24 h, after which the wound site was reimaged to monitor the healing process.

### Cell viability assay

CMECs were plated in 96-well plates and cultured until reaching approximately 80% confluence. Cell viability was evaluated via the 3-(4,5-dimethylthiazol-2-yl)-2,5-diphenyltetrazolium bromide (MTT) assay. Following the removal of the culture medium, MTT solution (0.01 mg/ml) was added, and cells were incubated at 37 °C for 4 h. The resulting formazan crystals were solubilized in dimethyl sulfoxide, and absorbance was recorded at 570 nm using a microplate spectrophotometer. Viability percentages were determined by normalizing the optical density of the H/R group to that of the control group, which was defined as 100% viability.

### Statistical analysis and reproducibility

All experiments were conducted with a minimum of 3 biological replicates, each comprising 3 technical replicates. Data are denoted as mean ± SD based on at least 3 independent experimental repeats. Statistical analyses were performed using GraphPad Prism software (version 9.2.0). For comparisons between the 2 groups, statistical significance was assessed using a 2-tailed unpaired *t* test. In analyses involving multiple groups with equal variances, significance was determined via one-way analysis of variance (ANOVA) followed by Tukey’s multiple comparisons test or 2-way ANOVA with a Bonferroni post hoc correction. For datasets exhibiting unequal variances, Brown–Forsythe and Welch ANOVA were employed, followed by Dunnett’s post hoc test. *P* values of <0.05 were considered statistically significant.

## Ethical Approval

The animal experiments were approved and performed in accordance with the guidelines of the animal care and use committee of Harbin Medical University (approval no.: HMUDQ20250820001).

## Data Availability

The mass spectrometry proteomics data have been deposited to the ProteomeXchange with the dataset identifier PXD066510. Additional data required from this study are available from the corresponding authors upon reasonable request.

## References

[1] McAlpine CS, Kiss MG, Rattik S, He S, Vassalli A, Valet C, Anzai A, Chan CT, Mindur JE, Kahles F, et al. Sleep modulates haematopoiesis and protects against atherosclerosis. Nature. 2019;566(7744):383–387. 30760925 10.1038/s41586-019-0948-2 PMC6442744

[2] Ai S, Zhang J, Zhao G, Wang N, Li G, So HC, Liu Y, Chau SW, Chen J, Tan X, et al. Causal associations of short and long sleep durations with 12 cardiovascular diseases: Linear and nonlinear Mendelian randomization analyses in UK Biobank. Eur Heart J. 2021;42(34):3349–3357. 33822910 10.1093/eurheartj/ehab170

[3] Jeddi S, Asl AN, Asgari A, Ghasemi A. The effect of sleep deprivation on cardiac function and tolerance to ischemia-reperfusion injury in male rats. Arq Bras Cardiol. 2016;106(1):41–48. 26559853 10.5935/abc.20150137 PMC4728594

[4] Ziegler KA, Ahles A, Dueck A, Esfandyari D, Pichler P, Weber K, Kotschi S, Bartelt A, Sinicina I, Graw M, et al. Immune-mediated denervation of the pineal gland underlies sleep disturbance in cardiac disease. Science. 2023;381(6655):285–290. 37471539 10.1126/science.abn6366

[5] Cappuccio FP, Cooper D, D’Elia L, Strazzullo P, Miller MA. Sleep duration predicts cardiovascular outcomes: A systematic review and meta-analysis of prospective studies. Eur Heart J. 2011;32(12):1484–1492. 21300732 10.1093/eurheartj/ehr007

[6] Wang J, Shao F, Yu QX, Ye L, Wusiman D, Wu R, Tuo Z, Wang Z, Li D, Cho WC, et al. The common hallmarks and interconnected pathways of aging, circadian rhythms, and cancer: Implications for therapeutic strategies. Research. 2025;8:0612. 40046513 10.34133/research.0612 PMC11880593

[7] Daghlas I, Dashti HS, Lane J, Aragam KG, Rutter MK, Saxena R, Vetter C. Sleep duration and myocardial infarction. J Am Coll Cardiol. 2019;74(10):1304–1314. 31488267 10.1016/j.jacc.2019.07.022 PMC6785011

[8] McAlpine CS, Kiss MG, Zuraikat FM, Cheek D, Schiroli G, Amatullah H, Huynh P, Bhatti MZ, Wong LP, Yates AG, et al. Sleep exerts lasting effects on hematopoietic stem cell function and diversity. J Exp Med. 2022;219(11): Article e20220081. 36129517 10.1084/jem.20220081 PMC9499822

[9] Herrmann J, Haude M, Lerman A, Schulz R, Volbracht L, Ge J, Schmermund A, Wieneke H, von Birgelen C, Eggebrecht H, et al. Abnormal coronary flow velocity reserve after coronary intervention is associated with cardiac marker elevation. Circulation. 2001;103(19):2339–2345. 11352881 10.1161/01.cir.103.19.2339

[10] Heusch G. Coronary microvascular obstruction: The new frontier in cardioprotection. Basic Res Cardiol. 2019;114(6):45. 31617010 10.1007/s00395-019-0756-8

[11] Niccoli G, Montone RA, Ibanez B, Thiele H, Crea F, Heusch G, Bulluck H, Hausenloy DJ, Berry C, Stiermaier T, et al. Optimized treatment of ST-elevation myocardial infarction. Circ Res. 2019;125(2):245–258. 31268854 10.1161/CIRCRESAHA.119.315344

[12] Kleinbongard P, Heusch G. A fresh look at coronary microembolization. Nat Rev Cardiol. 2022;19(4):265–280. 34785770 10.1038/s41569-021-00632-2 PMC8593642

[13] Heusch G. Myocardial ischaemia-reperfusion injury and cardioprotection in perspective. Nat Rev Cardiol. 2020;17(12):773–789. 32620851 10.1038/s41569-020-0403-y

[14] Piper HM, García-Dorado D, Ovize M. A fresh look at reperfusion injury. Cardiovasc Res. 1998;38(2):291–300. 9709390 10.1016/s0008-6363(98)00033-9

[15] Bei Y, Wang H, Liu Y, Su Z, Li X, Zhu Y, Zhang Z, Yin M, Chen C, Li L, et al. Exercise-induced miR-210 promotes cardiomyocyte proliferation and survival and mediates exercise-induced cardiac protection against ischemia/reperfusion injury. Research. 2024;7:0327. 38410280 10.34133/research.0327 PMC10895486

[16] Hausenloy DJ, Yellon DM. The mitochondrial permeability transition pore: Its fundamental role in mediating cell death during ischaemia and reperfusion. J Mol Cell Cardiol. 2003;35(4):339–341. 12689812 10.1016/s0022-2828(03)00043-9

[17] Pikwong F, Kamsarn J, Jarisarapurin W, Baipaywad P, Park H, Kumphune S. Cardiac cell membrane-coated nanoparticles as a potential targeted delivery system for cardiac therapy. Biomimetics. 2025;10(3):141. 40136795 10.3390/biomimetics10030141 PMC11940174

[18] Zhang H, Wang Y, Qu M, Li W, Wu D, Cata JP, Miao C. Neutrophil, neutrophil extracellular traps and endothelial cell dysfunction in sepsis. Clin Transl Med. 2023;13(1): Article e1170. 36629024 10.1002/ctm2.1170 PMC9832433

[19] Herre M, Cedervall J, Mackman N, Olsson AK. Neutrophil extracellular traps in the pathology of cancer and other inflammatory diseases. Physiol Rev. 2023;103(1):277–312. 35951483 10.1152/physrev.00062.2021 PMC9576172

[20] Mousset A, Lecorgne E, Bourget I, Lopez P, Jenovai K, Cherfils-Vicini J, Dominici C, Rios G, Girard-Riboulleau C, Liu B, et al. Neutrophil extracellular traps formed during chemotherapy confer treatment resistance via TGF-β activation. Cancer Cell. 2023;41(4):757–775.e10. 37037615 10.1016/j.ccell.2023.03.008 PMC10228050

[21] Zhang H, Liu L, Shen C, Jiang X, Liu J, Chen J, Senlei X, Mo Y. Lactate-induced mitochondrial calcium uptake 3 aggravates myocardial ischemia–reperfusion injury by promoting neutrophil extracellular trap formation. Research. 2025;8:0705. 40452820 10.34133/research.0705 PMC12123085

[22] Heusch G, Andreadou I, Bell R, Bertero E, Botker HE, Davidson SM, Downey J, Eaton P, Ferdinandy P, Gersh BJ, et al. Health position paper and redox perspectives on reactive oxygen species as signals and targets of cardioprotection. Redox Biol. 2023;67: Article 102894. 37839355 10.1016/j.redox.2023.102894 PMC10590874

[23] Chen C, Zhang H, Xie R, Wang Y, Ma Y. Gut microbiota aggravate cardiac ischemia-reperfusion injury via regulating the formation of neutrophils extracellular traps. Life Sci. 2022;303: Article 120670. 35640777 10.1016/j.lfs.2022.120670

[24] Thiam HR, Wong SL, Wagner DD, Waterman CM. Cellular mechanisms of NETosis. Annu Rev Cell Dev Biol. 2020;36:191–218. 32663035 10.1146/annurev-cellbio-020520-111016 PMC8499668

[25] Jankowski J, Floege J, Fliser D, Böhm M, Marx N. Cardiovascular disease in chronic kidney disease: Pathophysiological insights and therapeutic options. Circulation. 2021;143(11):1157–1172. 33720773 10.1161/CIRCULATIONAHA.120.050686 PMC7969169

[26] Chen L, Min J, Wang F. Copper homeostasis and cuproptosis in health and disease. Signal Transduct Target Ther. 2022;7(1):378. 36414625 10.1038/s41392-022-01229-y PMC9681860

[27] Garza NM, Swaminathan AB, Maremanda KP, Zulkifli M, Gohil VM. Mitochondrial copper in human genetic disorders. Trends Endocrinol Metab. 2023;34(1):21–33. 36435678 10.1016/j.tem.2022.11.001 PMC9780195

[28] Guengerich FP. Introduction to metals in biology 2018: Copper homeostasis and utilization in redox enzymes. J Biol Chem. 2018;293(13):4603–4605. 29425098 10.1074/jbc.TM118.002255 PMC5880126

[29] Qiao L, Lu Y, Liu B, Girault HH. Copper-catalyzed tyrosine nitration. J Am Chem Soc. 2011;133(49):19823–19831. 22046951 10.1021/ja206980q

[30] Yang N, Cao DF, Yin XX, Zhou HH, Mao XY. Lysyl oxidases: Emerging biomarkers and therapeutic targets for various diseases. Biomed Pharmacother. 2020;131: Article 110791. 33152948 10.1016/j.biopha.2020.110791

[31] Liu Y, Xiao Y, Liu J, Feng L, Kang YJ. Copper-induced reduction in myocardial fibrosis is associated with increased matrix metalloproteins in a rat model of cardiac hypertrophy. Metallomics. 2018;10(1):201–208. 29302675 10.1039/c7mt00165g

[32] Zhang S, Liu H, Amarsingh GV, Cheung CC, Hogl S, Narayanan U, Zhang L, McHarg S, Xu J, Gong D, et al. Diabetic cardiomyopathy is associated with defective myocellular copper regulation and both defects are rectified by divalent copper chelation. Cardiovasc Diabetol. 2014;13:100. 24927960 10.1186/1475-2840-13-100 PMC4070334

[33] Xiao Y, Wang T, Song X, Yang D, Chu Q, Kang YJ. Copper promotion of myocardial regeneration. Exp Biol Med. 2020;245(10):911–921. 10.1177/1535370220911604 PMC7268932 32148090

[34] Guo J, Bai Y, Liao J, Wang S, Han Q, Tang Z. Copper induces apoptosis through endoplasmic reticulum stress in skeletal muscle of broilers. Biol Trace Elem Res. 2020;198(2):636–643. 32080790 10.1007/s12011-020-02076-0

[35] Tsvetkov P, Coy S, Petrova B, Dreishpoon M, Verma A, Abdusamad M, Rossen J, Joesch-Cohen L, Humeidi R, Spangler RD, et al. Copper induces cell death by targeting lipoylated TCA cycle proteins. Science. 2022;375(6586):1254–1261. 35298263 10.1126/science.abf0529 PMC9273333

[36] Liu H, Chen A. Roles of sleep deprivation in cardiovascular dysfunctions. Life Sci. 2019;219:231–237. 30630005 10.1016/j.lfs.2019.01.006

[37] Schömig A, Richardt G. Cardiac sympathetic activity in myocardial ischemia: Release and effects of noradrenaline. Basic Res Cardiol. 1990;85(Suppl 1):9–30. 10.1007/978-3-662-11038-6_2 2091611

[38] Heusch G, Deussen A, Thämer V. Cardiac sympathetic nerve activity and progressive vasoconstriction distal to coronary stenoses: Feed-back aggravation of myocardial ischemia. J Auton Nerv Syst. 13(4):311–326. 4031366 10.1016/0165-1838(85)90020-7

[39] Heusch G. Alpha-adrenergic mechanisms in myocardial ischemia. Circulation. 1990;81(1):1–13. 1967557 10.1161/01.cir.81.1.1

[40] Heusch G, Baumgart D, Camici P, Chilian W, Gregorini L, Hess O, Indolfi C, Rimoldi O. Alpha-adrenergic coronary vasoconstriction and myocardial ischemia in humans. Circulation. 2000;101(6):689–694. 10673263 10.1161/01.cir.101.6.689

[41] Chen N, Guo L, Wang L, Dai S, Zhu X, Wang E. Sleep fragmentation exacerbates myocardial ischemia–reperfusion injury by promoting copper overload in cardiomyocytes. Nat Commun. 2024;15(1):3834. 38714741 10.1038/s41467-024-48227-y PMC11076509

[42] Schulz R, Kelm M, Heusch G. Nitric oxide in myocardial ischemia/reperfusion injury. Cardiovasc Res. 2004;61(3):402–413. 14962472 10.1016/j.cardiores.2003.09.019

[43] Timmers L, Pasterkamp G, de Hoog VC, Arslan F, Appelman Y, de Kleijn DP. The innate immune response in reperfused myocardium. Cardiovasc Res. 2012;94(2):276–283. 22266751 10.1093/cvr/cvs018

[44] Zuurbier CJ, Abbate A, Cabrera-Fuentes HA, Cohen MV, Collino M, De Kleijn DPV, Downey JM, Pagliaro P, Preissner KT, Takahashi M, et al. Innate immunity as a target for acute cardioprotection. Cardiovasc Res. 2019;115(7):1131–1142. 30576455 10.1093/cvr/cvy304 PMC6529915

[45] Dörge H, Neumann T, Behrends M, Skyschally A, Schulz R, Kasper C, Erbel R, Heusch G. Perfusion-contraction mismatch with coronary microvascular obstruction: Role of inflammation. Am J Physiol Heart Circ Physiol. 2000;279(6):H2587–H2592. 11087208 10.1152/ajpheart.2000.279.6.H2587

[46] Stakos DA, Kambas K, Konstantinidis T, Mitroulis I, Apostolidou E, Arelaki S, Tsironidou V, Giatromanolaki A, Skendros P, Konstantinides S, et al. Expression of functional tissue factor by neutrophil extracellular traps in culprit artery of acute myocardial infarction. Eur Heart J. 2015;36(22):1405–1414. 25660055 10.1093/eurheartj/ehv007 PMC4458286

[47] Nakazawa D, Kumar SV, Marschner J, Desai J, Holderied A, Rath L, Kraft F, Lei Y, Fukasawa Y, Moeckel GW, et al. Histones and neutrophil extracellular traps enhance tubular necrosis and remote organ injury in ischemic AKI. J Am Soc Nephrol. 2017;28(6):1753–1768. 28073931 10.1681/ASN.2016080925 PMC5461800

[48] Heusch G. Vagal cardioprotection in reperfused acute myocardial infarction. JACC Cardiovasc Interv. 2017;10(15):1521–1522. 28797428 10.1016/j.jcin.2017.05.063

[49] Lieder HR, Kleinbongard P, Skyschally A, Hagelschuer H, Chilian WM, Heusch G. Vago-splenic axis in signal transduction of remote ischemic preconditioning in pigs and rats. Circ Res. 2018;123(10):1152–1163. 30359199 10.1161/CIRCRESAHA.118.313859 PMC7304918

[50] Heusch G, Kleinbongard P. The spleen in ischaemic heart disease. Nat Rev Cardiol. 2025;22(7):497–509. 39743566 10.1038/s41569-024-01114-x

[51] Lieder HR, Paket U, Skyschally A, Rink AD, Baars T, Neuhäuser M, Kleinbongard P, Heusch G. Vago-splenic signal transduction of cardioprotection in humans. Eur Heart J. 2024;45(34):3164–3177. 38842545 10.1093/eurheartj/ehae250

[52] Rosen SD, Murphy K, Leff AP, Cunningham V, Wise RJ, Adams L, Coats AJ, Camici PG. Is central nervous system processing altered in patients with heart failure? Eur Heart J. 2004;25(11):952–962. 15172467 10.1016/j.ehj.2004.03.025

[53] Zhang M, Qiu H, Mao L, Wang B, Li N, Fan Y, Weng P, Hu S, Dong X, Qin X, et al. Ammonium tetrathiomolybdate triggers autophagy-dependent NRF2 activation in vascular endothelial cells. Cell Death Dis. 2022;13(8):733. 36008391 10.1038/s41419-022-05183-z PMC9411162

[54] Zhang L, Hoffman JA, Ruoslahti E. Molecular profiling of heart endothelial cells. Circulation. 2005;112(11):1601–1611. 16144998 10.1161/CIRCULATIONAHA.104.529537

[55] Mu Q, Yao K, Syeda MZ, Wan J, Cheng Q, You Z, Sun R, Zhang Y, Zhang H, Lu Y, et al. Neutrophil targeting platform reduces neutrophil extracellular traps for improved traumatic brain injury and stroke theranostics. Adv Sci. 2024;11(21): Article e2308719. 10.1002/advs.202308719 PMC11151022 38520727

[56] Carter JR, Fonkoue IT, Greenlund IM, Schwartz CE, Mokhlesi B, Smoot CA. Sympathetic neural responsiveness to sleep deprivation in older adults: Sex differences. Am J Physiol Heart Circ Physiol. 2019;317(2):H315–H322. 31149842 10.1152/ajpheart.00232.2019 PMC6732487

[57] Wang Z, Liang X, Lu Y, Jiang T, Aji T, Aimulajiang K, Sun H, Zhang L, Zhou X, Tang B, et al. Insomnia promotes hepatic steatosis in rats possibly by mediating sympathetic overactivation. Front Physiol. 2021;12: Article 734009. 34630154 10.3389/fphys.2021.734009 PMC8497715

[58] Grassi G, Mark A, Esler M. The sympathetic nervous system alterations in human hypertension. Circ Res. 2015;116(6):976–990. 25767284 10.1161/CIRCRESAHA.116.303604 PMC4367954

[59] Shen MJ, Zipes DP. Role of the autonomic nervous system in modulating cardiac arrhythmias. Circ Res. 2014;114(6):1004–1021. 24625726 10.1161/CIRCRESAHA.113.302549

[60] Oyama J, Node K. Sympathetic nerve activity and endothelial function. Hypertens Res. 2014;37(12):1035–1036. 25231255 10.1038/hr.2014.142

[61] Zeng W, Pirzgalska RM, Pereira MM, Kubasova N, Barateiro A, Seixas E, Lu YH, Kozlova A, Voss H, Martins GG, et al. Sympathetic neuro-adipose connections mediate leptin-driven lipolysis. Cell. 2015;163(1):84–94. 26406372 10.1016/j.cell.2015.08.055 PMC7617198

[62] Takase B, Akima T, Satomura K, Ohsuzu F, Mastui T, Ishihara M, Kurita A. Effects of chronic sleep deprivation on autonomic activity by examining heart rate variability, plasma catecholamine, and intracellular magnesium levels. Biomed Pharmacother. 2004;58(Suppl 1):S35–S39. 15754837 10.1016/s0753-3322(04)80007-6

[63] Dou H, Kotini A, Liu W, Fidler T, Endo-Umeda K, Sun X, Olszewska M, Xiao T, Abramowicz S, Yalcinkaya M, et al. Oxidized phospholipids promote NETosis and arterial thrombosis in LNK(SH2B3) deficiency. Circulation. 2021;144(24):1940–1954. 34846914 10.1161/CIRCULATIONAHA.121.056414 PMC8663540

[64] Carai P, González LF, Van Bruggen S, Spalart V, De Giorgio D, Geuens N, Martinod K, Jones EAV, Heymans S. Neutrophil inhibition improves acute inflammation in a murine model of viral myocarditis. Cardiovasc Res. 2023;118(17):3331–3345. 35426438 10.1093/cvr/cvac052 PMC9847559

[65] Hao P, Li H, Zhou L, Sun H, Han J, Zhang Z. Serum metal ion-induced cross-linking of photoelectrochemical peptides and circulating proteins for evaluating cardiac ischemia/reperfusion. ACS Sens. 2022;7(3):775–783. 35293731 10.1021/acssensors.1c02305

[66] Chevion M, Jiang Y, Har-El R, Berenshtein E, Uretzky G, Kitrossky N. Copper and iron are mobilized following myocardial ischemia: Possible predictive criteria for tissue injury. Proc Natl Acad Sci USA. 1993;90(3):1102–1106. 8430081 10.1073/pnas.90.3.1102 PMC45819

[67] Toscano CM, Filetti FM, Almenara CCP, Fioresi M, Vassallo DV. Copper exposure for 30 days at a daily dose twice the recommended increases blood pressure and cardiac contractility. Life Sci. 2022;300: Article 120579. 35489564 10.1016/j.lfs.2022.120579

[68] Zhong G, Li L, Li Y, Ma F, Liao J, Li Y, Zhang H, Pan J, Hu L, Tang Z. Cuproptosis is involved in copper-induced hepatotoxicity in chickens. Sci Total Environ. 2023;866: Article 161458. 36621474 10.1016/j.scitotenv.2023.161458

[69] Hartwig C, Zlatic SA, Wallin M, Vrailas-Mortimer A, Fahrni CJ, Faundez V. Trafficking mechanisms of P-type ATPase copper transporters. Curr Opin Cell Biol. 2019;59:24–33. 30928671 10.1016/j.ceb.2019.02.009 PMC6726579

[70] Hatori Y, Lutsenko S. The role of copper chaperone Atox1 in coupling redox homeostasis to intracellular copper distribution. Antioxidants. 2016;5(3):25. 27472369 10.3390/antiox5030025 PMC5039574

[71] Heusch G. Myocardial ischemia/reperfusion: Translational pathophysiology of ischemic heart disease. Med. 2024;5(1):10–31. 38218174 10.1016/j.medj.2023.12.007

[72] Wiśniewski JR, Zougman A, Nagaraj N, Mann M. Universal sample preparation method for proteome analysis. Nat Methods. 2009;6(5):359–362. 19377485 10.1038/nmeth.1322

[73] Kessenbrock K, Krumbholz M, Schönermarck U, Back W, Gross WL, Werb Z, Gröne HJ, Brinkmann V, Jenne DE. Netting neutrophils in autoimmune small-vessel vasculitis. Nat Med. 2009;15(6):623–625. 19448636 10.1038/nm.1959 PMC2760083

[74] Yang L, Liu Q, Zhang X, Liu X, Zhou B, Chen J, Huang D, Li J, Li H, Chen F, et al. DNA of neutrophil extracellular traps promotes cancer metastasis via CCDC25. Nature. 2020;583(7814):133–138. 32528174 10.1038/s41586-020-2394-6

[75] Miller EW, Zeng L, Domaille DW, Chang CJ. Preparation and use of Coppersensor-1, a synthetic fluorophore for live-cell copper imaging. Nat Protoc. 2006;1(2):824–827. 17406313 10.1038/nprot.2006.140

