# Supplementary material for: Neuromodulation and Copper Chelation Reverse Sleep Fragmentation-Aggravated Myocardial Ischemia–Reperfusion Injury by Targeting NET-Induced Endothelial Cuproptosis
Source: Research (Wash D C). 2026 May 8;9:1266. doi: 10.34133/research.1266 (PMC13153460; doi:10.34133/research.1266)
Supplement: Supplementary 1 — Tables S1 and S2 Figs. S1 to S10 [file research.1266.f1.docx]

**Supplemental Information**

**Table S1 Primary antibodies information**

| **Company** | **Name** | **CAT.NO** | **Dilution ratio (Application)** |
| --- | --- | --- | --- |
| Proteintech | CD31 | 28083-1-AP | 1:2000 (WB); 1:200 (IF) |
| Proteintech | CXCR2 | 85144-5-RR | 1:2000 (WB) |
| SANTA | DLAT | SC-271534 | 1:200 (WB) |
| Abclonal | DLST | A13297 | 1:1000 (WB) |
| Proteintech | FDX1 | 12592-1-AP | 1:2000 (WB) |
| Abclonal | HSP70 | A23457 | 1:2000 (WB) |
| Proteintech | Atox1 | 84305-1-RR | 1:1000 (WB) |
| Abclonal | ly6G | A22270 | 1:200 (IF) |
| Abclonal | MPO | A22900 | 1:200 (IF) |
| Abcam | citH3 | Ab281584 | 1:200 (IF) |
| SANTA | ATP7A | sc-376467 | 1:200 (IF) |
| ZSGB-BIO | GAPDH | TA-08 | 1:2000 (WB) |

**Table S2 Primer sequence of RT-PCR, and gene knockdown sequence**

| **Gene name** | **Forward (5'-3')** | **Reverse (5'-3')** |
| --- | --- | --- |
| IL-1β | AGCTTCAAATCTCGCAGCAG | TCTCCACAGCCACAATGAGT |
| IL-6 | GACTGATGCTGGTGACAACC | AGACAGGTCTGTTGGGAGTG |
| IL-18 | ACTTTGGCCGACTTCACTGT | CCTCGAACACAGGCTGTCTT |
| Atox1 | TCTCCGTGGACATGACCTGTGAG | AGAGTCGATGCAGACCTTCTTGTTG |
| **Knockdown genes** | **Primer Sequence 5'-3'** | |
| Atox1 | GCAUCGACUCUGAGCACAGCUTT  AGCUGUGCUCAGAGUCGAUGCTT | |

**Supplemental figures and figure legends:**


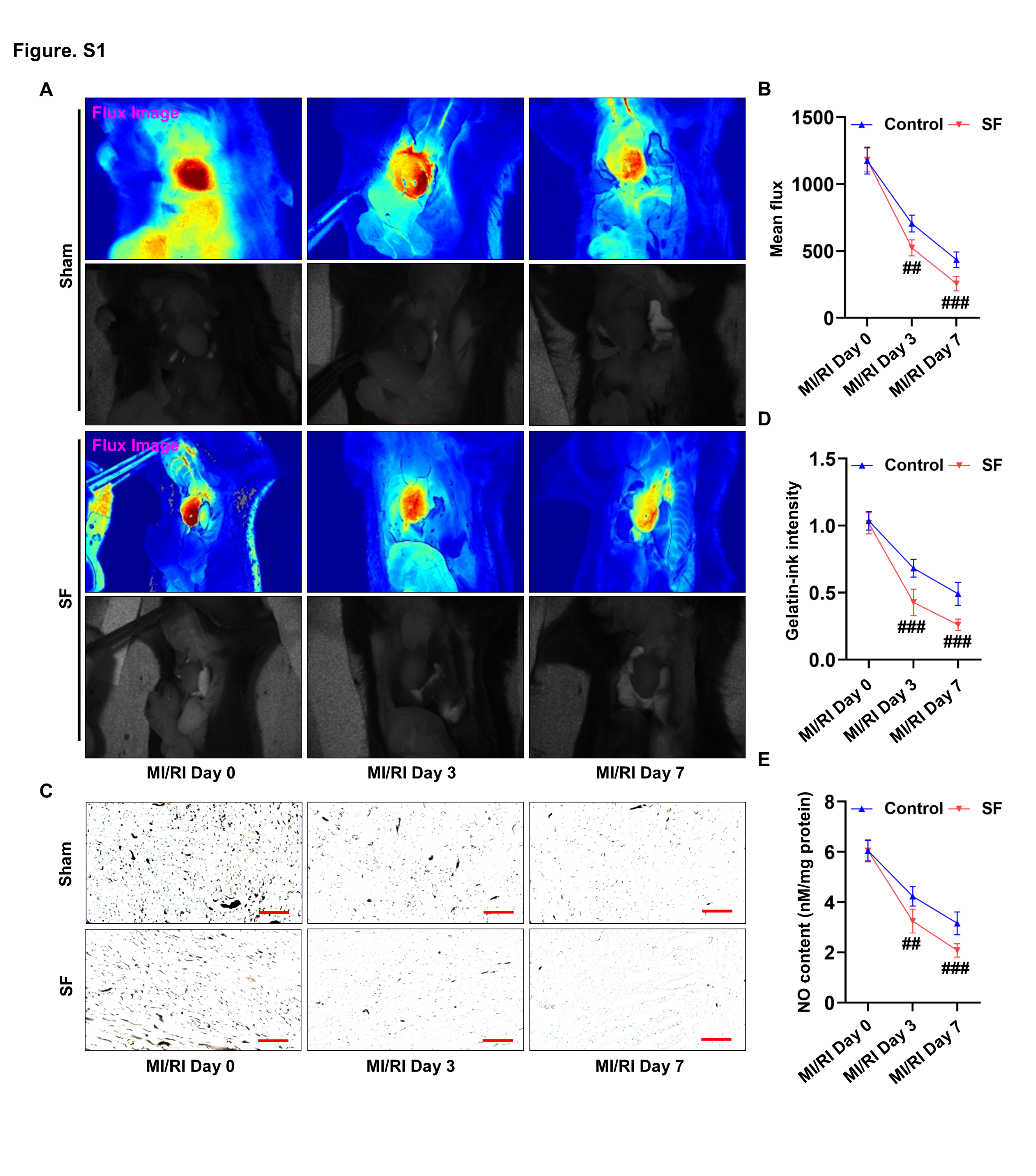


**Supplementary figure S1 Chronic SF exacerbated cardiac microvascular injury in MI/RI mice**

**(A-B)** The LASCA technique was used to assess the flow velocity in the infarct area at different reperfusion days (n = 6 per group). **(C-D)** Hearts were injected with gelatin-ink, and samples were observed via microscope on the 3rd and 7rd days of reperfusion (n = 6 per group). **(E)** Statistical analysis of nitric oxide content in myocardial tissues (n = 6 per group). Data are presented as mean ± SD. ^##^*P* < 0.01 vs. MI/RI Day 0 group; ^###^*P* < 0.001 vs. MI/RI Day 0 group. Scale bars: 50 μm.


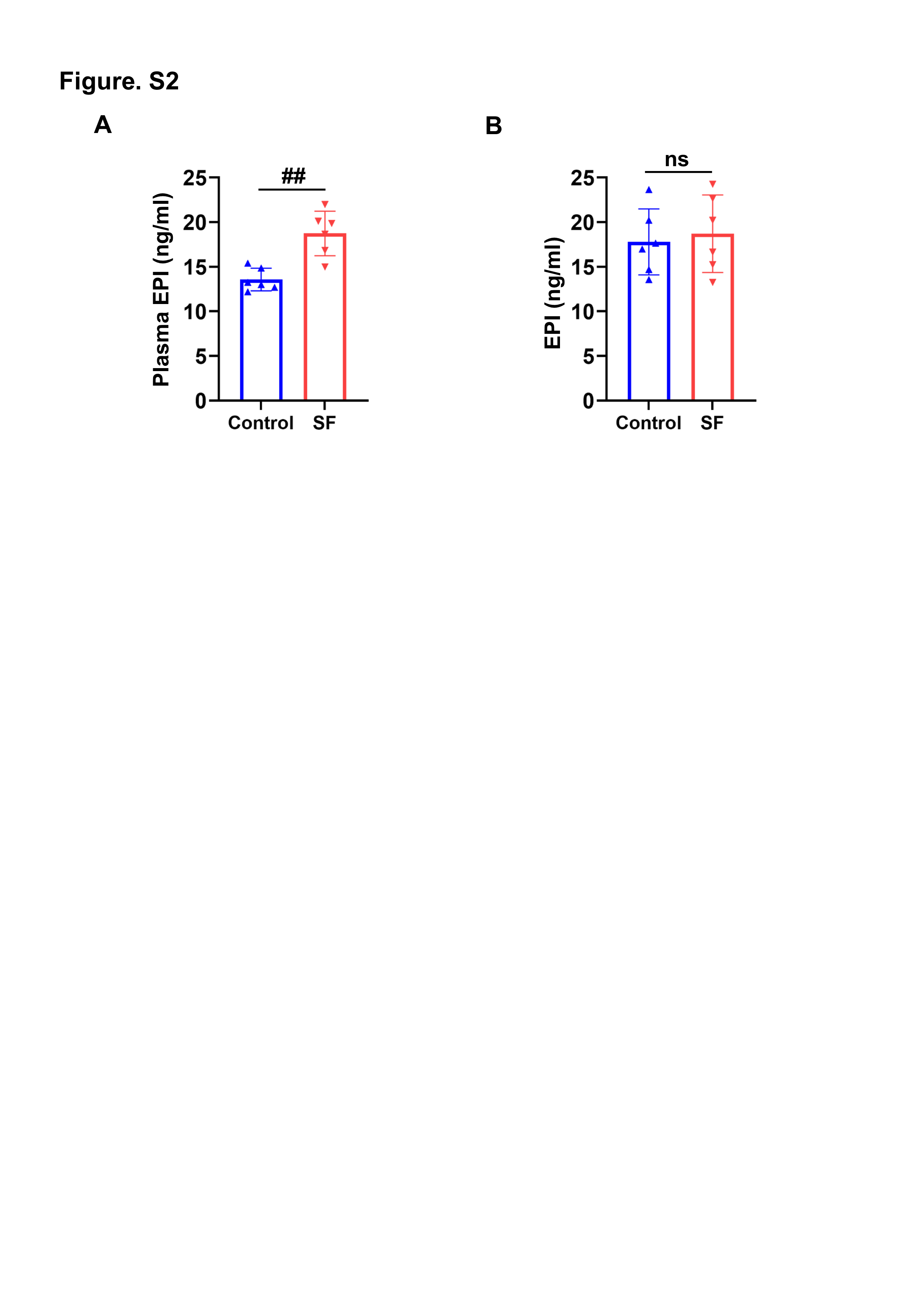


**Supplementary figure S2 Fragmented sleep led to sympathetic hyperactivity**

**(A)** ELISA was performed to detect epinephrine (EPI) in the plasma of mice with SF and control mice (n = 6 per group). **(B)** ELISA was performed to detect EPI in the cardiac tissue homogenate (n = 6 per group). Data are presented as mean ± SD. ^##^*P* < 0.01 vs. Control group. ns: not significant.


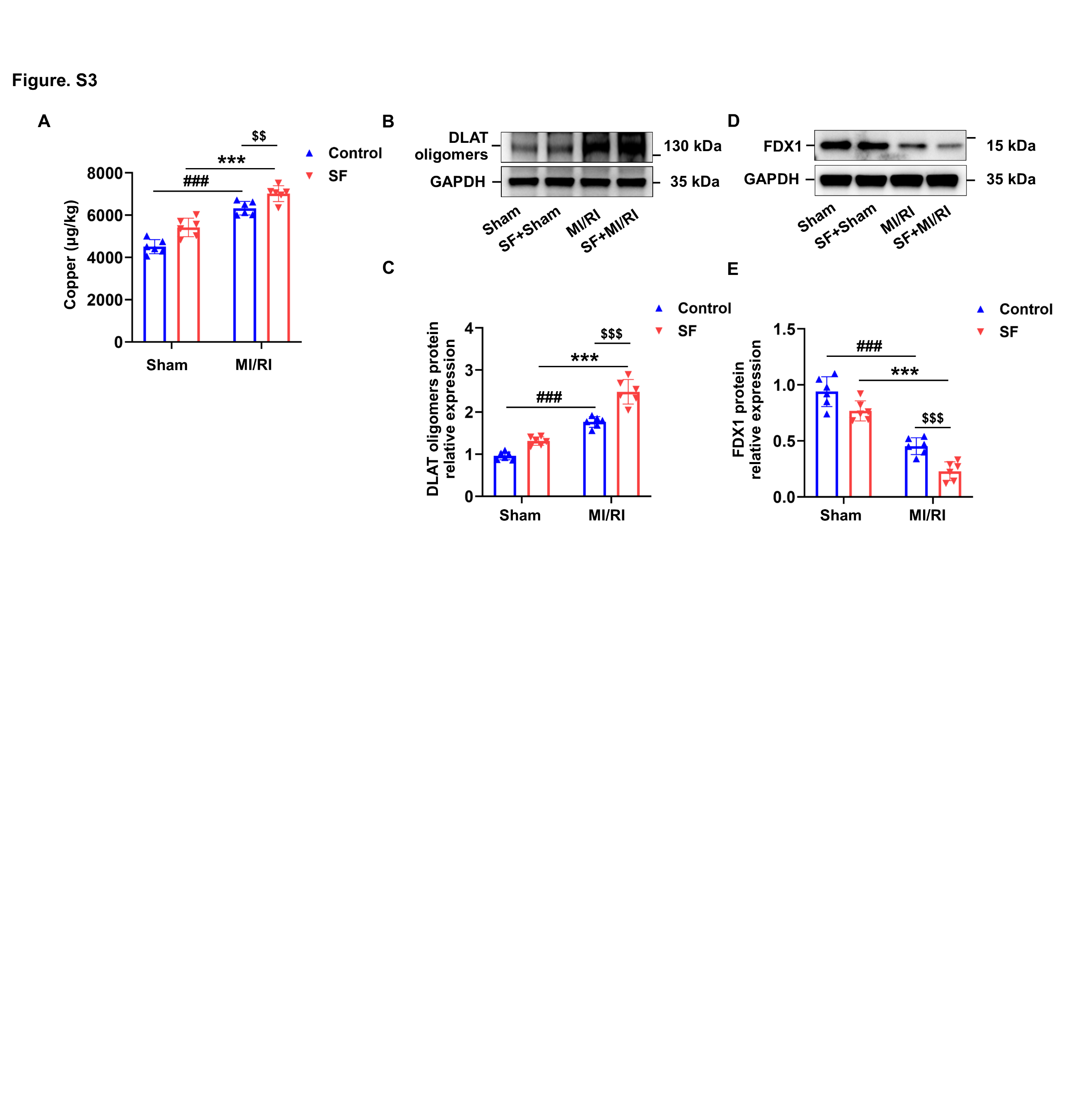


**Supplementary figure S3 SF exacerbated copper overload after MI/RI**

**(A)** Myocardial copper ions in mice with SF and MI/RI was detected via inductively coupled plasma‒mass spectrometry (ICP‒MS) (n = 6 per group). **(B-E)** Validation of the levels of DLAT oligomers and iron-sulfur cluster proteins by western blotting (n = 6 per group). Data are presented as mean ± SD. ^###^*P* < 0.001 vs. Sham group; ^***^*P* < 0.001 vs. SF+Sham group; ^$$^*P* < 0.01 vs. MI/RI group; ^$$$^*P* < 0.001 vs. MI/RI group.


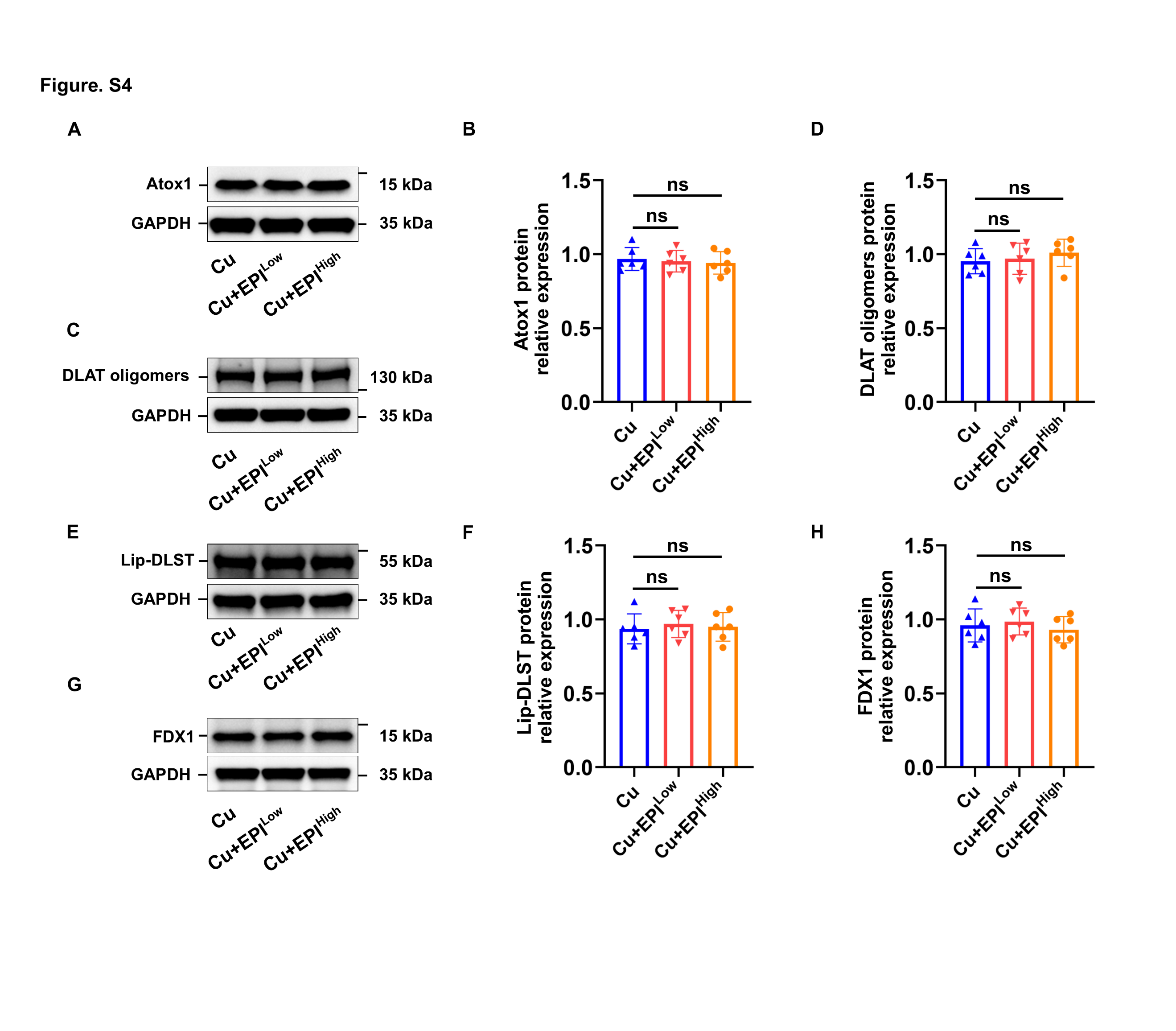


**Supplementary figure S4 EPI had no direct effect on cuproptosis in CMECs**

**(A-B)** The protein level of Atox1 was analyzed by western blotting in CMECs treated with 10 µM CuCl_2_ for 24 h in the presence of 1 µM or 10 µM EPI (n = 6 per group). **(C-H)** Validation of the levels of DLAT oligomers, lipoylated proteins and iron-sulfur cluster proteins by western blotting (n = 6 per group). Data are presented as mean ± SD. ns: not significant.

**
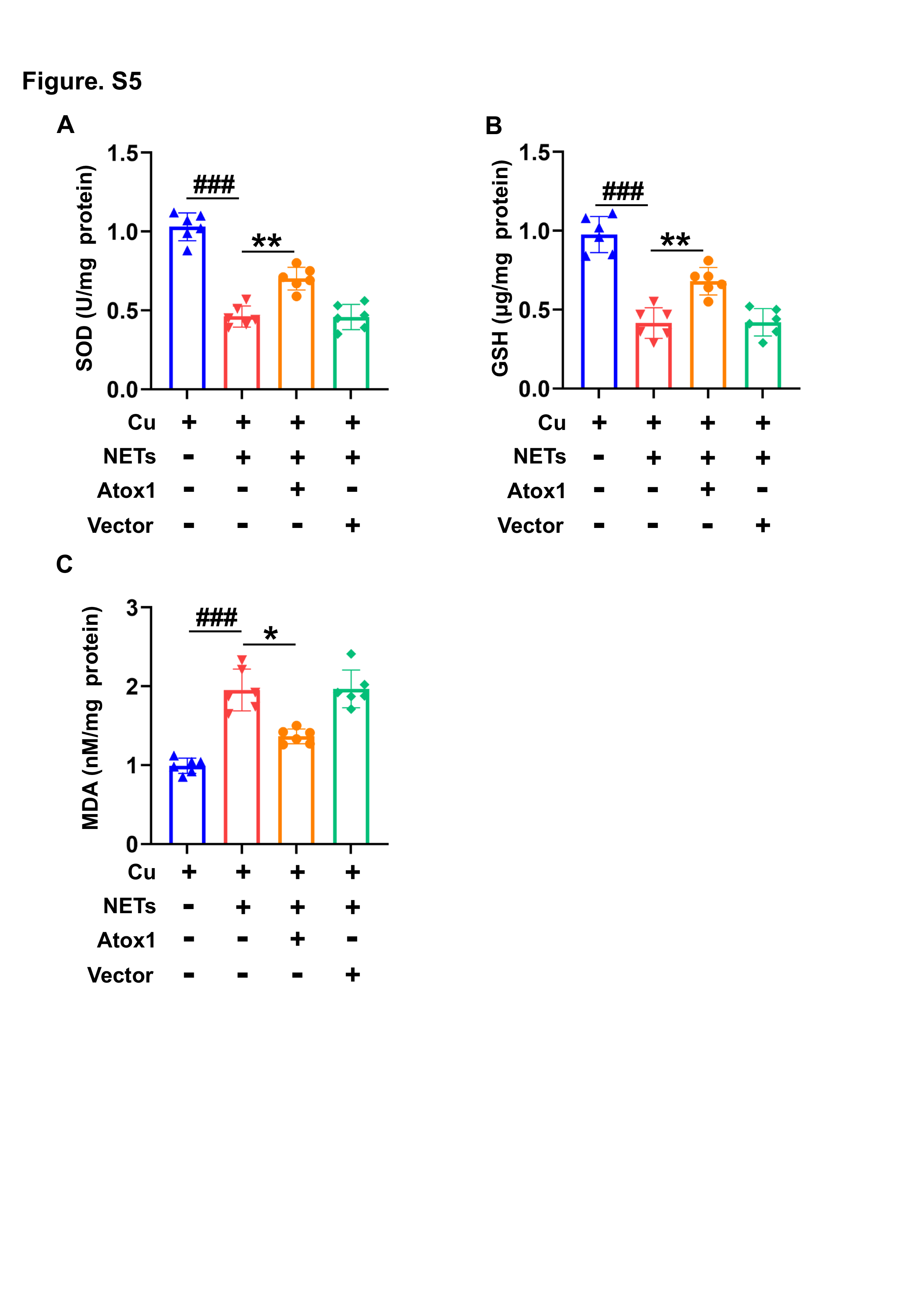
**

**Supplementary figure S5 Atox1 overexpression inhibited oxidative stress induced by NETs**

**(A)** The level of superoxide dismutase (SOD) (n = 6 per group). **(B)** The level of glutathione (GSH) (n = 6 per group). **(C)** The level of malondialdehyde (MDA) (n = 6 per group). Data are presented as mean ± SD. ^###^*P* < 0.001 vs. CMECs+Cu group; ^*^*P* < 0.05 vs. CMECs+Cu+NETs group; ^**^*P* < 0.01 vs. CMECs+Cu+NETs group.


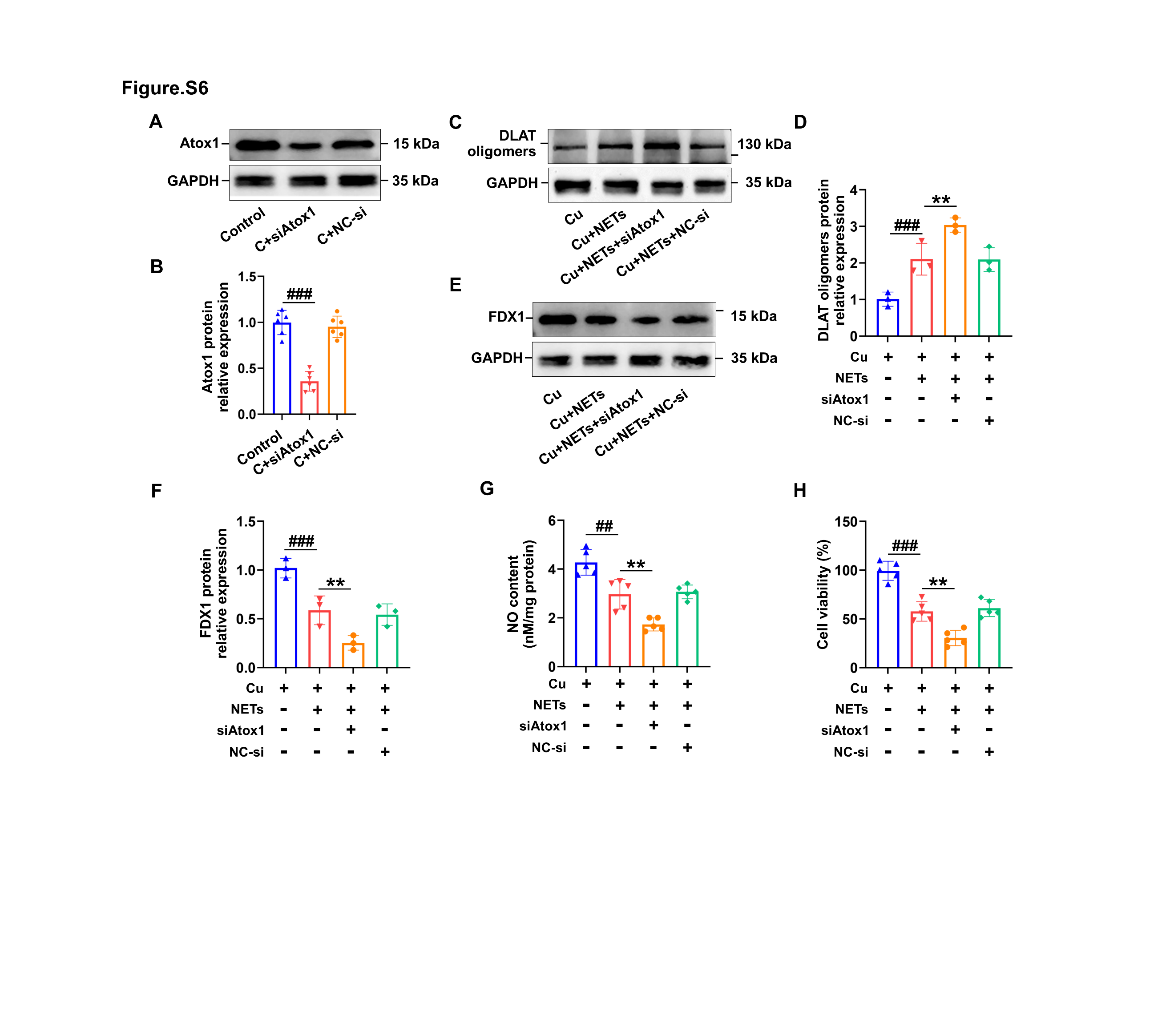


**Supplementary figure S6 Knockdown of Atox1 aggravated cuproptosis in CMECs induced by NETs**

**(A-B)** The protein level of Atox1 was analyzed by western blotting (n = 6 per group). ^###^*P* < 0.001 vs. Control group. **(C-F)** The protein level of DLAT oligomers and FDX1 were analyzed by western blotting (n = 3 per group). **(G)** Statistical analysis of NO content (n = 5 per group). **(H)** Cell viability was evaluated using the CCK-8 assay (n = 5 per group). Data are presented as mean ± SD. ^##^*P* < 0.01 vs. CMECs+Cu group; ^###^*P* < 0.001 vs. CMECs+Cu group; ^**^*P* < 0.01 vs. CMECs+Cu+NETs group.


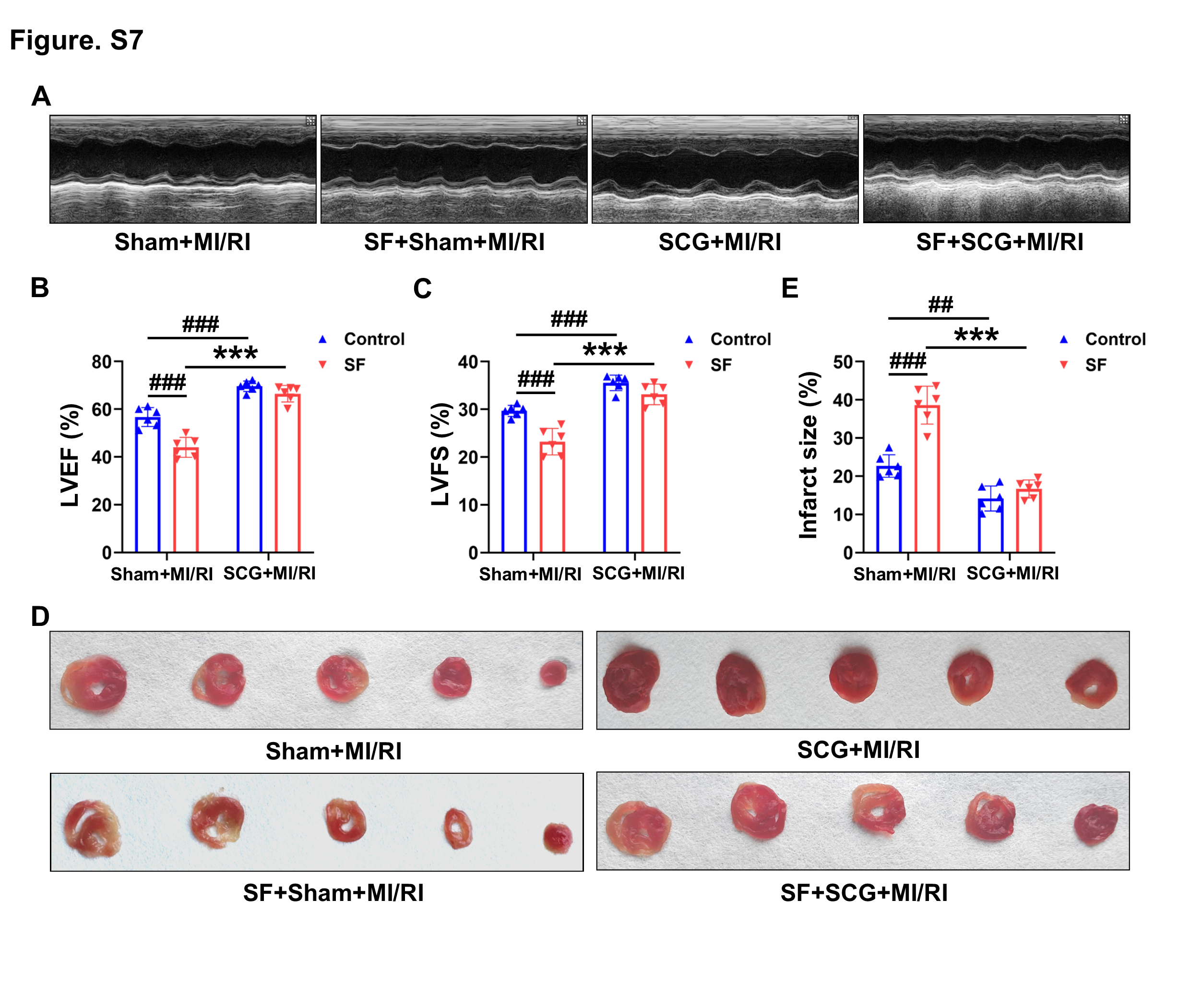


**Supplementary figure S7 SCG attenuated MI/RI in mice with SF**

**(A)** Representative M-mode echocardiographic changes showed that SCG attenuated the impaired cardiac function in mice with SF and MI/RI. **(B-C)** Statistical analysis of left ventricular ejection fraction (LVEF) and left ventricular fractional shortening (LVFS) (n = 6 per group). **(D)** Representative TTC staining of the myocardium. **(E)** The percentage of infarct area was determined by TTC staining (n = 6 per group). Data are presented as mean ± SD. ^##^*P* < 0.01 vs. Sham+MI/RI group; ^###^*P* < 0.001 vs. Sham+MI/RI group; ^***^*P* < 0.001 vs. SF+Sham+MI/RI group.


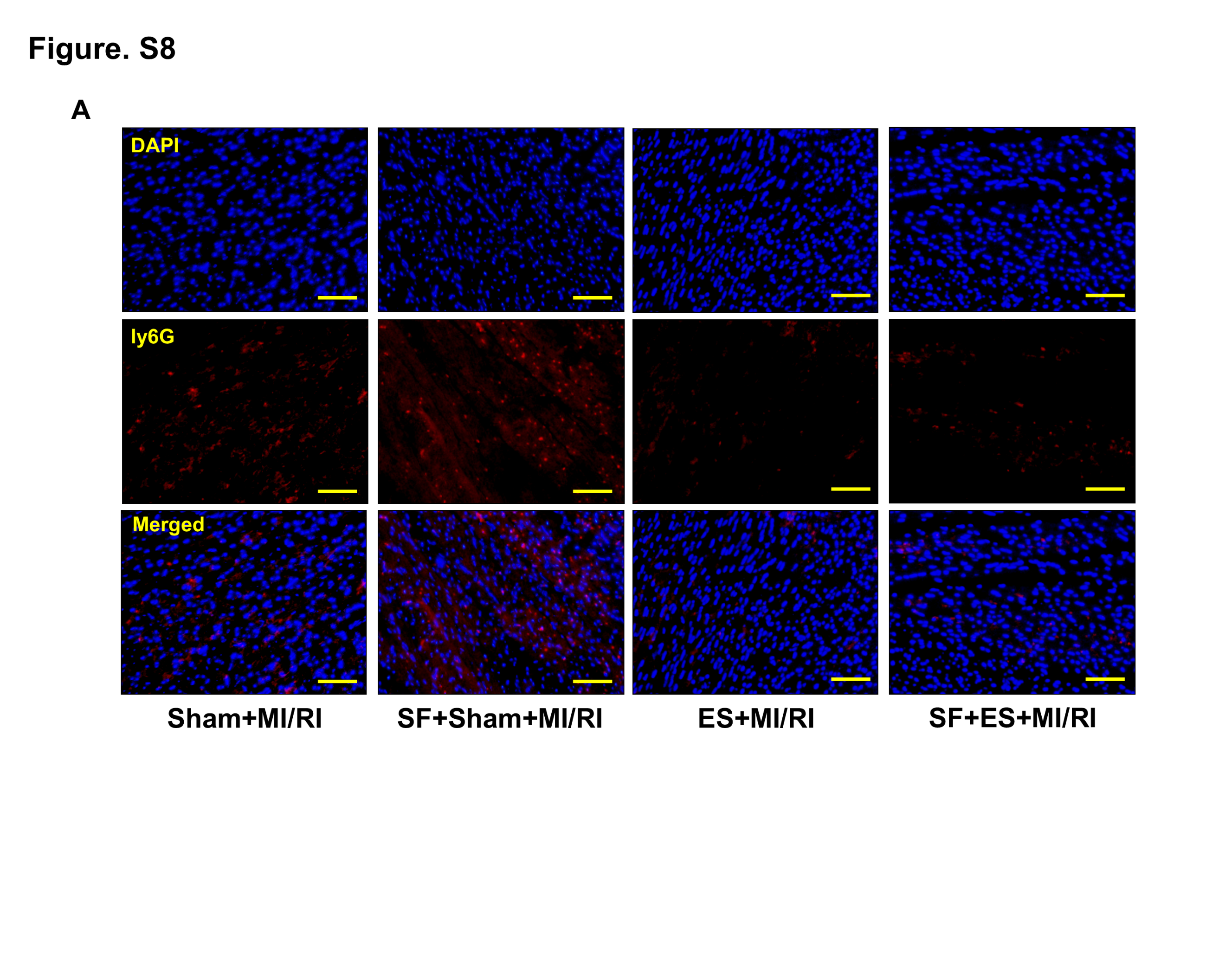


**Supplementary figure S8 ES reduced the chemotaxis of neutrophils towards the heart** **in mice with SF**

**(A)** Representative images of immunofluorescence staining of ly6G in myocardial tissues from each group (red: ly6G, blue: DAPI) (n = 6 per group). Scale bars: 50 μm.


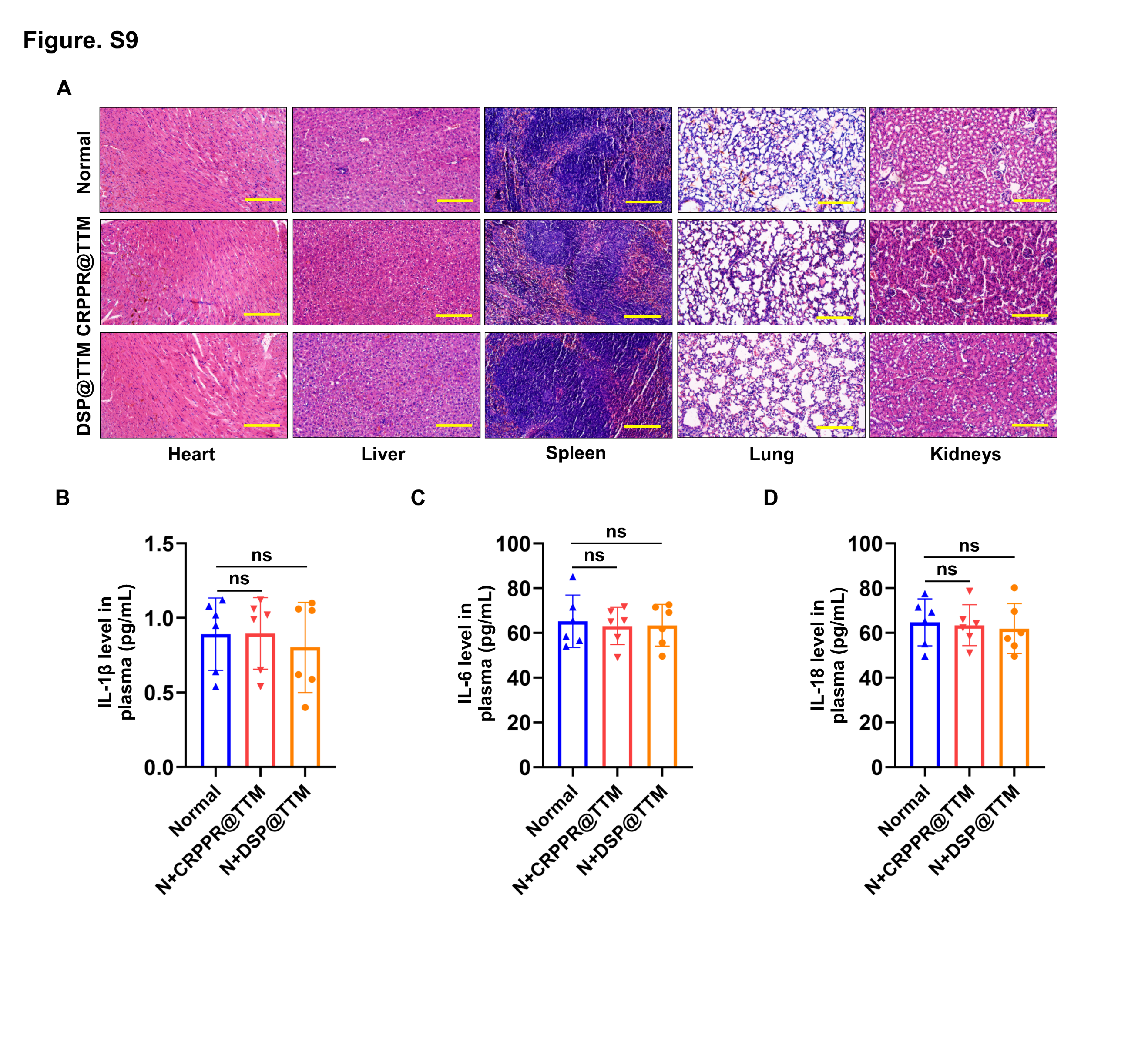


**Supplementary figure S9 Safety assessment of CRPPR@TTM and DSP@TTM**

**(A)** Histology characteristics of major organs of CRPPR@TTM or DSP@TTM treated healthy rat were detected by HE staining (n = 6 per group). **(B-D)** Concentrations of IL-1β, IL-6 and IL-18 in these mice were measured by ELISA (n = 6 per group). Data are presented as mean ± SD. ns: not significant. Scale bars: 100 μm.

**
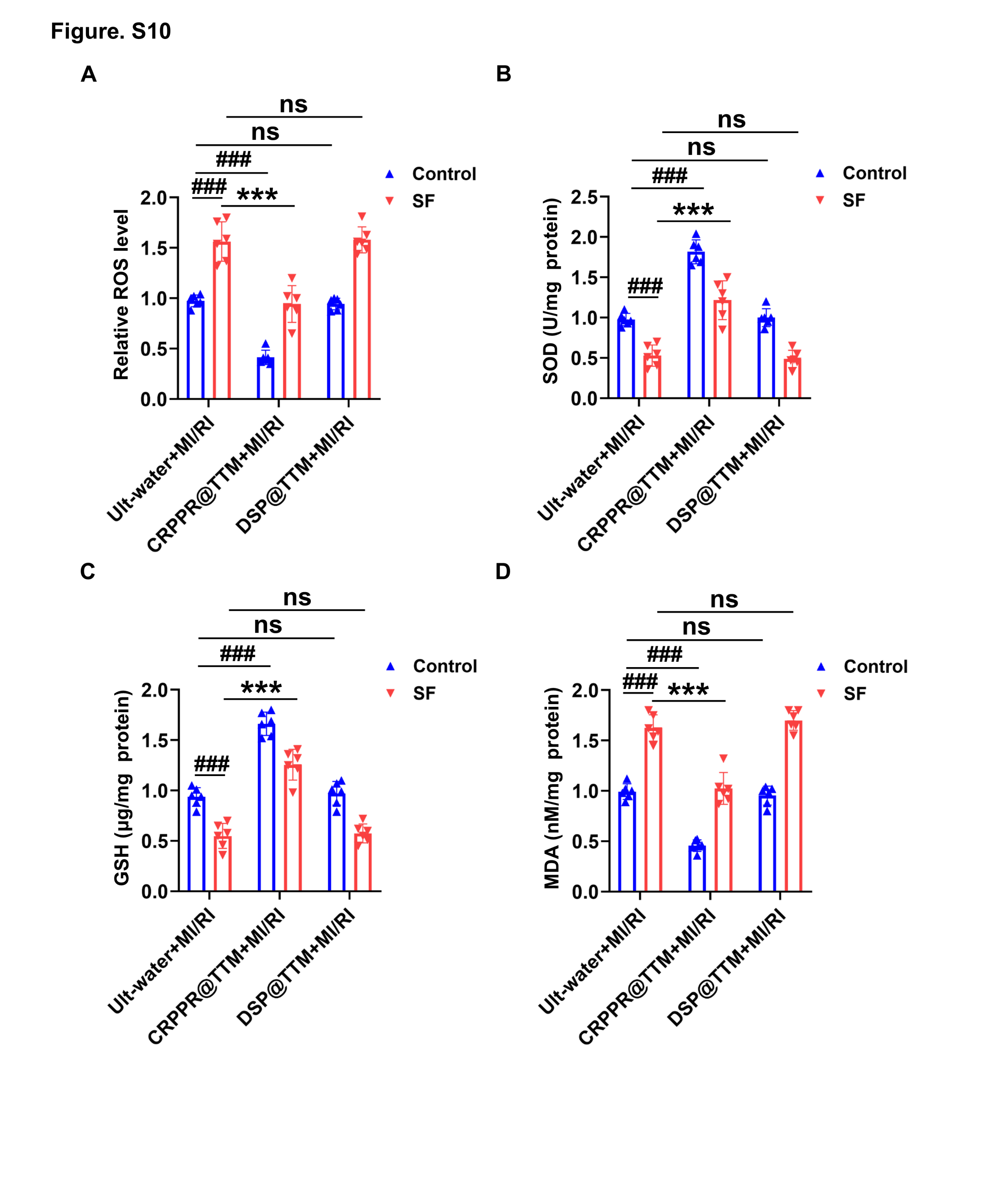
**

**Supplementary figure S10 CRPPR@TTM protected CMECs against oxidative stress in MI/RI with SF**

**(A-D)** ELISA was performed to detect the level of ROS, SOD, GSH and MDA (n = 6 per group). Data are presented as mean ± SD. ^###^*P* < 0.001 vs. Ult-water+MI/RI group; ^***^*P* < 0.001 vs. Ult-water+SF+MI/RI group. ns: not significant.
